# Unique macrophage phenotypes activated by BMP signaling in breast cancer bone metastases

**DOI:** 10.1172/jci.insight.168517

**Published:** 2024-01-09

**Authors:** Claire L. Ihle, Desiree M. Straign, Johana A. Canari, Kathleen C. Torkko, Kathryn L. Zolman, Elizabeth E. Smith, Philip Owens

**Affiliations:** 1 Department of Pathology, University of Colorado Anschutz Medical Campus, Aurora, Colorado, USA.; 2 Case Western Reserve University, Cleveland, Ohio, USA.; 3 Research Service, Department of Veterans Affairs, Eastern Colorado Health Care System, Aurora, Colorado, USA.

**Keywords:** Immunology, Oncology, Breast cancer, Innate immunity

## Abstract

Metastatic breast cancer (mBC) tissue in bone was systematically profiled to define the composition of the tumor microenvironment. Gene expression identified a high myeloid signature of patients with improved survival outcomes. Bone metastases were profiled by spatial proteomics to examine myeloid populations within the stroma that correlated with macrophage functions. Single-cell spatial analysis uncovered macrophage activation in the stroma of mBC bone lesions. Matched BC patient samples of primary breast tumor and bone metastasis tissues were compared for gene expression in the bone, where bone morphogenetic protein 2 (*BMP2*) was most significantly upregulated. Immune cell changes from breast to bone demonstrated a loss of lymphoid cells but a consistent population of macrophages. BMP-activated macrophages were increased uniquely in bone. Bone marrow–derived macrophage activation coupled with BMP inhibition increased inflammatory responses. Using experimental mouse models of mBC bone metastasis and trained immunity, we found that BMP inhibition restricts progression of metastases early in the macrophage activation state but not after tumors were established in the bone. This study revealed unique myeloid BMP activation states that are distinctly integrated with bone metastases.

## Abstract 2

Spatial macrophage phenotypes determine how both the location and timing of activation can influence metastatic breast cancer in bone.

## Graphical abstract

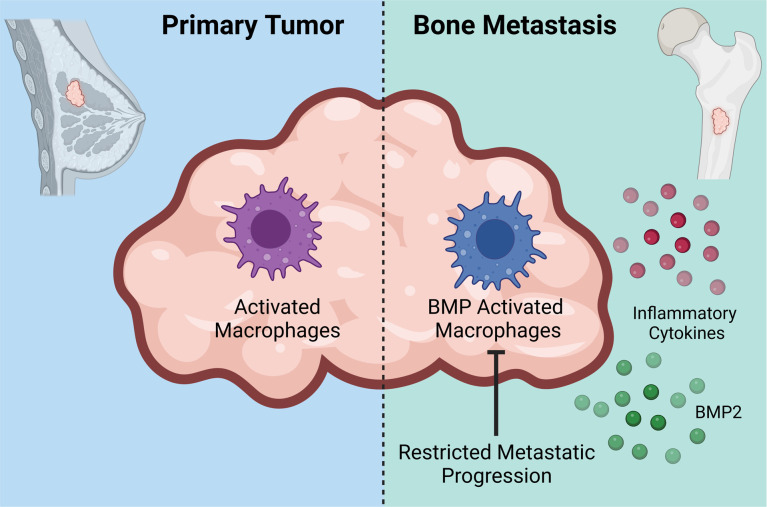

## Introduction

Breast cancer (BC) accounts for almost one-third of cancer diagnoses for women in the United States annually (1). Patients with metastatic BC (mBC) with distant disease experience a markedly diminished clinical outlook, with a 5-year relative survival rate of only 29% (1). The most common mBC site is the bone, with bone metastases being found in 70% of patients with mBC (2). Currently, bone metastases are not universally biopsied, and collected tissue is not extensively profiled. Patients with mBC in the bone have limited therapeutic options, none of which are curative. Thus, there is an unmet need to identify markers unique to mBC bone lesions that could be developed into targeted therapies to help prolong patient survival.

The bone is enriched with diverse myeloid cells that regulate bone remodeling and hematopoiesis, and this results in a unique niche for mBC (3). The tumor microenvironment (TME) of mBC overall has lower immune cell surveillance but higher immunosuppressive macrophages compared with primary BC tumors (4). Immune checkpoint blockade therapy has only benefited a limited number of patients with mBC; thus, investigating other drivers of the mBC bone TME could help identify therapeutic targets that reprogram immune cells to be antitumorigenic.

Macrophages in the TME can exhibit heterogeneous and multifunctional states, and approaches to functionally reprogram macrophages to be antitumorigenic are the target of therapeutic development (5). Trained immunity in myeloid cells is a potential targeting framework for cancer therapies (6). Myeloid cells in the TME undergo reprogramming to be antitumorigenic due to trained immunity stimulus in multiple mouse models (7, 8). Trained immunity programming occurs in the myeloid progenitor cells of the bone marrow, suggesting that trained immunity may play a distinct role in mBC bone lesions (9).

Unique to the bone microenvironment are bone morphogenetic proteins (BMPs), which are regulators of differentiation and control bone mineralization as well as hematopoietic stem cell homeostasis (10, 11). BMP signaling exhibits context-dependent roles in cancer, acting as a tumor promoter or tumor suppressor (12). Our previous work has determined that BMP signaling in myeloid cells supports myeloid progenitors in the bone marrow and in the prostate cancer TME (13). BMP signaling in the prostate cancer TME drives immunosuppressive and protumorigenic macrophages to promote tumor growth. Inhibition of BMP signaling in a mouse model of BC also revealed reduced metastasis to the lungs and reprogrammed myeloid cells in the TME to be antitumorigenic (14).

The goal of this study was to survey how mBC lesions in bone have a unique molecular and cellular phenotype. These findings in archival bone with mBC demonstrate the utility of high dimensional molecular and cellular diagnostic phenotyping for a previously difficult tissue to assess that has not been sufficiently studied. This investigation revealed myeloid phenotypes in mBC patient bone samples with distinct myeloid activation and BMP signaling. Mouse models revealed antitumorigenic myeloid memory functions were altered by inhibition of BMP signaling and restricted metastatic progression of mBC bone lesions. These findings suggest that a temporal specific BMP modulation may enhance therapeutic options to treat mBC bone lesions by reprogramming the TME.

## Results

### The TME landscape of metastatic BC in bone.

To investigate the cellular and molecular characteristics of the mBC bone TME, a cohort of archival decalcified formalin-fixed and paraffin-embedded (FFPE) mBC bone lesions was collected from patients at the University of Colorado Cancer Center. This mBC bone cohort currently consists of 21 patient samples, with most patients exhibiting estrogen receptor–positive (ER^+^) primary tumors and bone metastases as well as osteolytic bone pathology (Supplemental Table 1; supplemental material available online with this article; https://doi.org/10.1172/jci.insight.168517DS1).

**Figure 1:**
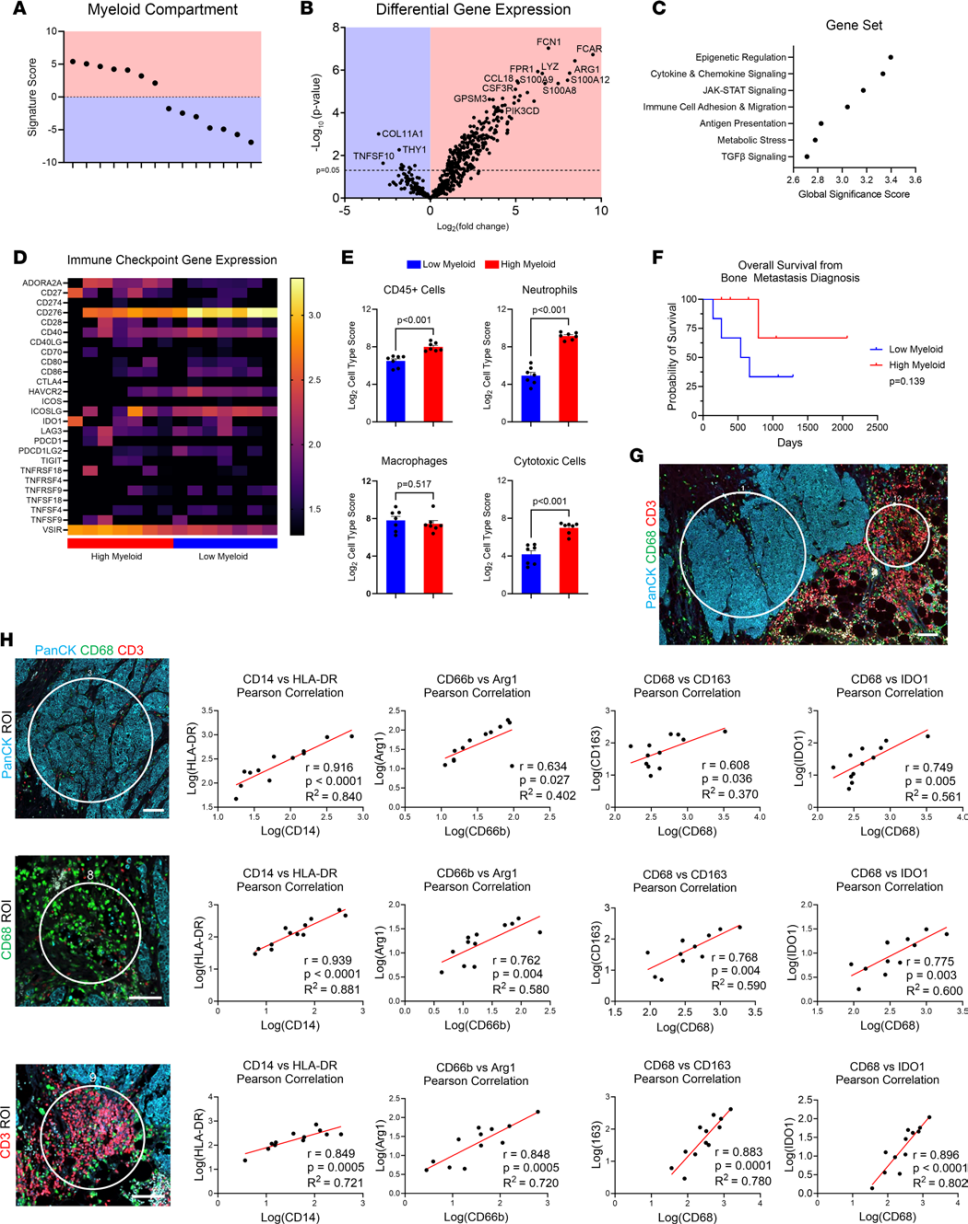
The diverse immune microenvironment of human breast cancer in bone. (**A**) Myeloid compartment signature scores from PanCancer IO 360 gene expression analysis of archival FFPE mBC patient bone metastases (*n* = 14). Cohort separated into samples exhibiting high myeloid compartment gene expression (*n* = 7, red region) and samples exhibiting low myeloid compartment gene expression (*n* = 7, blue region). (**B**) Volcano plot of differential gene expression analysis of high myeloid compartment patients compared with the baseline of low myeloid compartment patients. Genes upregulated in high myeloid compartment patients are shown on the right (red region), and genes upregulated in low myeloid compartment patients are shown on the left (blue region). (**C**) Undirected gene set enrichment pathway analysis of high versus low myeloid compartment patient gene expression. Global significance scores greater than 1 revealed pathways significantly upregulated in high myeloid compartment patient samples. (**D**) Heatmap of immune checkpoint gene expression from mBC bone metastases. (**E**) Cell type gene signature scores for low myeloid compartment and high myeloid compartment patient samples. Data are presented as mean ± SEM. Statistical values determined by Student’s *t* test. (**F**) Kaplan-Meier curve of overall survival from mBC bone metastasis diagnosis to death in patient cohort based on high or low myeloid compartment gene expression. Statistical value determined by log-rank test. (**G**) Representative image of GeoMx Digital Spatial Profiling of FFPE archival mBC patient bone metastases (*n* = 12). Each patient was stamped with 4 PanCK ROIs, 4 CD68 ROIs, and 4 CD3 ROIs. Scale bar: 100 μM. (**H**) Correlations of myeloid marker protein expression and functional proteins in PanCK, CD68, and CD3 ROIs from mBC bone samples (*n* = 12). *R*^2^ value for simple linear regression reported. Statistical analysis determined by Pearson Correlation *r* value and *P* value. Scale bars: 100 μM.

To analyze the transcriptional landscape of mBC in bone, bulk RNA was isolated from scroll sections of decalcified FFPE mBC bone tissue. Probe-based bulk gene expression analysis with the NanoString human IO 360 nCounter panel was selected to profile the immune TME. Of our 21 patient samples analyzed with the nCounter platform, 14 samples yielded sufficient RNA quantity and quality to pass experimental quality control checks for bulk gene expression analysis. The 14 samples had distinct separation based on high (*n* = 7) and low (*n* = 7) myeloid compartment signature scores (Figure 1A). Differential gene expression analysis between the high and low myeloid compartment gene signature samples revealed unique myeloid function genes in the patients with high myeloid expression (high myeloid patients) (Figure 1B, Supplemental Figure 1A, and Supplemental Table 2). Myeloid genes enriched in the high myeloid compartment patient group included the bacteriolytic enzyme lysozyme (*LYZ*), inflammatory genes *S100A8* and *S100A12*, *CCL18*, and the immunosuppressive gene arginase 1 (*ARG1*). Inflammatory gene pathways — including cytokine and chemokine signaling, JAK/STAT signaling, and immune cell adhesion and migration — were upregulated in the high myeloid gene signature patient group (Figure 1C). Patients with mBC in the bone have exhibited poor clinical responses to immune checkpoint inhibitors (15). Unsurprisingly, most immune checkpoint genes were not highly enriched in the mBC patient bone samples (Figure 1D). Clinically actionable targets including *CTLA4* and *CD274* (PD-L1) had low expression in all bone samples. The most abundant immune checkpoint targets observed were *CD276* (B7-H3) and *VSIR* (VISTA). The immune checkpoint genes *VSIR* (VISTA) and *CD27* were significantly enriched in the high myeloid compartment patient samples compared with the low myeloid compartment patient samples (Supplemental Table 2). Immune cell gene signature scores revealed that CD45^+^ immune cells, neutrophils, and cytotoxic cells were significantly enhanced in the high myeloid gene signature patients compared with the low myeloid patients (Figure 1E). Surprisingly, macrophage gene scores were not distinguishable in the high myeloid gene signature patients. However, T cells and CD8^+^ cytotoxic T cells were also significantly enriched in the high myeloid patient samples (Supplemental Figure 1B).

To investigate how the TME of mBC bone metastases affect patient clinical outcome, the overall survival of patients was investigated. The high myeloid gene signature patients demonstrated a strong trend in prolonged overall survival from their bone metastasis diagnosis compared with the low myeloid gene signature patients (Figure 1F). Due to the limited sample size of our cohort, the survival benefit for patients with mBC with a high myeloid signature was not statistically significant, with a *P* value of 0.139. However, there is a clear separation in patient survival, since the probability of survival for the high myeloid patients (*n* = 7) was 66.6% three years after bone metastasis diagnosis, while the low myeloid patients (*n* = 7) only had a 33.3% probability of survival. This result demonstrates that the bone TME affects mBC patient outcome.

To determine the spatially restricted immune cell landscape of the TME in mBC bone, regional proteomic analysis was performed. mBC bone tissue contains an abundance of stromal cells surrounding the pancytokeratin (PanCK^+^) mBC tumor cells, including CD68^+^ cells such as macrophages and CD3^+^ cells such as T cells (Supplemental Figure 2, A–C). NanoString GeoMx Digital Spatial Profiling (DSP) was used to determine the regional proteomic characteristics of the PanCK, CD68, and CD3 enriched regions of the TME in mBC patient bone lesions. From our cohort of 21 mBC bone metastases, 12 samples had adequate tissue quantity and quality for analysis with the GeoMx DSP platform. Regions of interest (ROIs) were selected on the patient samples, with 4 PanCK-enriched ROIs, 4 CD68-enriched ROIs, and 4 CD3-enriched ROIs selected for each patient (Figure 1G). Both macrophages and bone resorbing macrophage-derived osteoclasts express CD68; thus, CD68-enriched ROIs were selected for CD68^+^ cells, including macrophages and excluding larger multinucleated CD68^+^ osteoclasts (16). Protein expression was measured in these distinct ROIs for the 12 patients with mBC in the bone marrow (Supplemental Tables 3–5). Protein expression correlation analysis was performed for myeloid cell markers and functional phenotypes in the 3 TME ROIs (Figure 1H). The myeloid cell marker CD14 positively correlated with the MHC-II protein HLA-DR, with the strongest correlation found in the CD68 ROIs. The CD66b neutrophil marker was positively correlated with the immunosuppressive protein Arg1, which is a marker for tumor-associated neutrophils (17). Interestingly, CD66b and Arg1 had the strongest positive correlation in the CD3 ROIs. Positive correlations were also observed with the macrophage marker CD68, the immunosuppressive M2-like macrophage marker CD163, and the immune checkpoint protein IDO1. The correlation between CD68 and CD163, as well as CD68 and IDO1, was strongest in the CD3 ROIs. These correlations indicate that myeloid cells exhibit diverse inflammatory and immunosuppressive phenotypes. All myeloid cell markers exhibited the weakest correlation coefficients in the PanCK ROIs of mBC in bone, while the CD68- and CD3-enriched stromal ROIs had the strongest myeloid cell correlations. The finding of strongest correlations in the stromal ROIs indicates that myeloid cells are enriched in the stroma of the bone TME rather than in the tumor. These findings demonstrate that heterogeneous myeloid cells are present in bone metastases and exhibit unique spatial phenotypes in the bone TME.

### Spatial phenotyping of immune cells in BC patient bone metastases.

**Figure 2:**
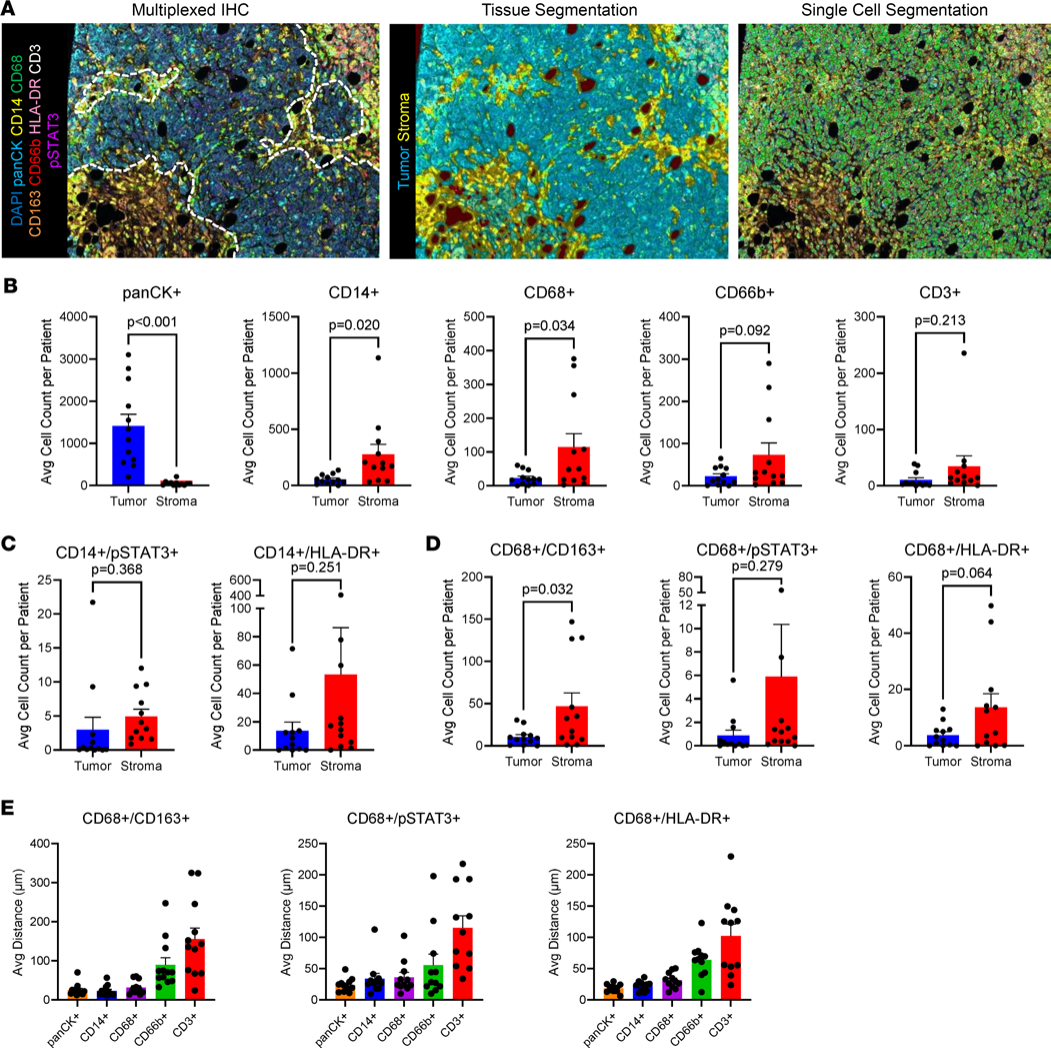
Single-cell spatial proteomic immune cell composition in human breast cancer bone metastases. (**A**) Polaris mIHC representative images of spectral unmixing, tissue segmentation, and single-cell segmentation of mBC bone metastasis samples (*n* = 12). Dotted white line indicates tumor-stroma boundary. (**B**) Cellular phenotype counts in bone metastasis tumor tissue segments and stromal tissue segments. (**C**) Myeloid cell phenotype counts in bone metastasis tumor tissue segments and stromal tissue segments. (**D**) Macrophage phenotype counts in bone metastasis tumor tissue segments and stromal tissue segments. (**E**) Nearest-neighbor analysis of the distance between macrophage cell phenotypes and other cell populations in mBC bone metastases. Data are presented as mean ± SEM. Statistical values determined by Student’s *t* test (**B**–**D**).

To expand the investigation of myeloid cell functions in the bone TME, findings from the bulk gene expression and regional spatial analysis were expanded to an investigation of single-cell protein expression. Multiplexed IHC (mIHC) single-cell analysis was performed on mBC bone metastasis patient samples with the Polaris platform. Twelve patient samples were analyzed from the cohort of 21 mBC bone tissues based on selection for tissue quality and quantity. mIHC-stained images underwent analysis with spectral unmixing, tissue segmentation for tumor and stroma, single-cell segmentation, and cell phenotyping with InForm (Figure 2A). Spatial analysis of cell phenotypes revealed that CD14^+^ myeloid cells and CD68^+^ macrophages were significantly enriched in the stromal tissue of bone metastases rather than the tumor tissue (Figure 2B and Supplemental Table 6). CD66b^+^ neutrophils exhibited a trend toward enrichment in the stromal tissue segment of mBC bone lesions compared with the tumor tissue segment. CD3^+^ T cells did not exhibit altered abundance in the tumor versus stromal tissue segments. Analysis of complex myeloid phenotypes showed CD14^+^/pSTAT3^+^ myeloid cells with inflammatory signaling, and CD14^+^/HLA-DR^+^ antigen-expressing myeloid cells were found both in the tumor and stroma tissue segments of bone metastases (Figure 2C). More complex macrophage phenotype analysis showed CD68^+^/pSTAT3^+^ inflammatory signaling macrophages were present both in the tumor and stroma, while CD68^+^/CD163^+^ immunosuppressive M2-like macrophages and CD68^+^/HLA-DR^+^ proinflammatory antigen-presenting macrophages more frequently populated the stroma (Figure 2D). Nearest-neighbor analysis revealed that both immunosuppressive and proinflammatory macrophages are nearest PanCK^+^ tumor cells, CD14^+^ myeloid cells, and other CD68^+^ macrophages and are farthest from CD66b^+^ neutrophils and CD3^+^ T cells in the TME of bone metastases (Figure 2E). This analysis demonstrated that mBC bone metastases exhibit a predominantly myeloid cell–excluded TME, with macrophages enriched in the peritumoral region of the stroma.

### Comparing distinct immune microenvironments of the breast tumor and bone metastasis.

**Figure 3:**
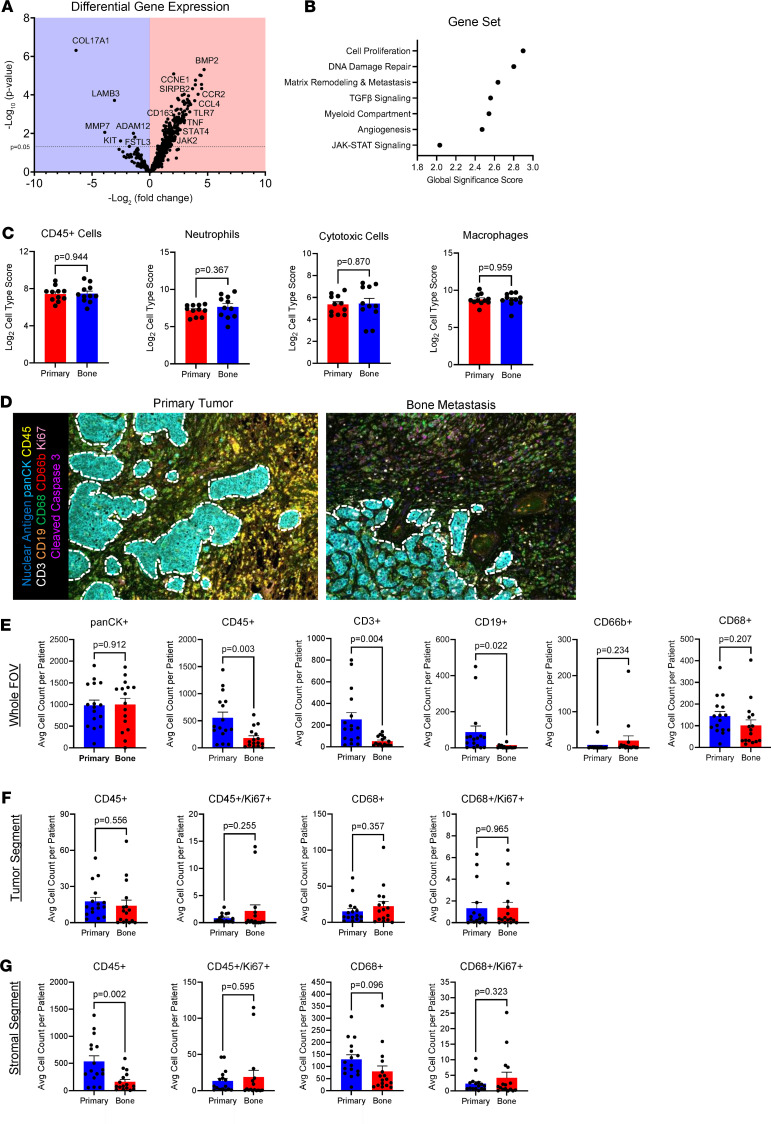
Comparative analysis of human breast cancer primary tumor and bone metastasis immune microenvironment. (**A**) Volcano plot of differential gene expression analysis from nCounter IO 360 gene expression analysis of mBC bone metastases compared with the baseline of matched primary tumors (*n* = 11). Genes upregulated in bone metastases are shown on the right (red region), and genes upregulated in primary tumors are shown on the left (blue region). (**B**) Undirected gene set enrichment pathway analysis of bone metastases versus primary tumor gene expression. Global significance scores greater than 1 revealed pathways significantly upregulated in bone metastases compartment–matched primary BC tumors. (**C**) Cell type gene signature scores for primary tumors and bone lesions. (**D**) Polaris mIHC representative image of spectrally unmixed mBC patient primary tumor and matched bone metastasis (*n* = 16). Dotted white line indicates tumor-stroma boundary. (**E**) Cellular phenotype counts in primary tumor and bone metastasis whole FOV images. (**F**) Cellular phenotype counts in the tumor tissue segments of primary tumor and bone metastasis samples. (**G**) Cellular phenotype counts in the stromal tissue segments of primary tumor and bone metastasis samples. Data are presented as mean ± SEM. Statistical values determined by Student’s *t* test (**C** and **E**–**G**).

To determine if the myeloid phenotypes observed in mBC bone lesions were due to a patient’s breast tumor phenotype or the unique TME of the bone, the TMEs from matched patient primary tumors and bone metastasis tissues were compared (Supplemental Table 1). From our mBC bone sample cohort, we acquired the matched primary breast tumor FFPE tissues for 16 of our patients. We isolated bulk RNA from matched primary tumor and bone metastasis FFPE scrolls from our 16 patients; then, nCounter IO 360 bulk gene expression analysis was performed. From the 16 matched primary tumor and bone metastasis patient samples run, 11 matched patient samples passed quality control analytical measures on the nCounter platform. Differential gene expression analysis comparing the 11 primary tumors with the matched bone metastases revealed that myeloid inflammatory genes and BMP signaling genes were the most significantly enriched in the bone metastases (Figure 3A, Supplemental Figure 3A, and Supplemental Table 7). The BMP ligand *BMP2* was the top upregulated gene in bone metastases, which has been shown to be secreted by macrophages, tumor-associated macrophages, and BC cells (18–20). Myeloid inflammatory genes *CCL4*, *TLR7*, and *TNFA* were also significantly upregulated. The immune checkpoint genes *ADORA2A*, *CD86* (B7-2), and *HAVCR2* (TIM3) were significantly increased in mBC bone metastases compared with matched primary tumors. Pathway analysis showed TGF-β signaling, myeloid compartment, and inflammatory JAK/STAT signaling were higher in bone metastases compared with matched primary tumors (Figure 3B). Immune cell populations based on gene signatures revealed no differences between the matched primary tumors and bone metastases (Figure 3C and Supplemental Figure 3B). These results suggest that the bone TME of mBC can alter myeloid functions, with unique macrophages populating bone metastases.

To examine the distinctions in immune cell populations between primary breast tumors and bone metastases with single-cell protein levels, a Polaris mIHC panel was developed. Analysis of tumor and stroma tissue segmentation, single-cell segmentation, and phenotypes was performed on the same cohort of matched primary BC and mBC bone samples, with 16 patients analyzed in total (Figure 3D and Supplemental Table 8). Analysis of whole field of view (FOV) images showed that primary breast tumors had significantly greater CD45^+^ immune cells, CD3^+^ T cells, and CD19^+^ B cells compared with the bone metastases (Figure 3E). However, CD66b^+^ neutrophil and CD68^+^ macrophage counts were unchanged between the primary tumor and bone metastasis FOVs. In tumor tissue segments, CD45^+^ immune cell and CD68^+^ macrophage infiltration, as well as Ki67 expression for proliferation, was not significantly altered in the primary tumors and bone metastases (Figure 3F). Stromal tissue segments in the primary tumors had higher CD45^+^ immune cell infiltration, but CD68^+^ macrophage infiltration was not significantly altered (Figure 3G). Ki67^+^ proliferating CD45^+^ immune cells and CD68^+^ macrophages were not altered in the stromal tissue segments of primary tumors and bone metastases. These findings determined that bone metastases exhibit a TME with overall decreased immune cells in whole FOV images and stromal segments. Macrophage counts were similar, yet macrophage inflammatory genes were globally enriched in the bone TME. These results suggest that macrophage activation states, rather than macrophage number, may be driving the distinct TME of mBC in bone.

### Altered macrophage activation states in breast tumor and bone metastases.

**Figure 4:**
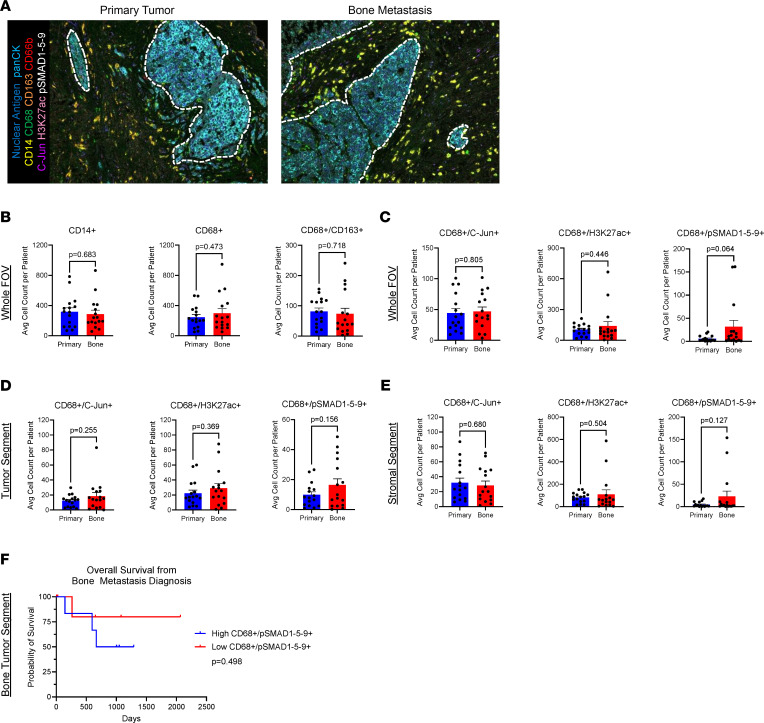
Altered macrophage activation states in human breast cancer primary tumors and bone metastases. (**A**) Polaris mIHC representative image of spectrally unmixed mBC patient primary tumor and matched bone metastasis (*n* = 16). Dotted white line indicates tumor-stroma boundary. (**B**) Myeloid cell phenotype counts in primary tumor and bone metastasis whole FOV images. (**C**) Macrophage phenotype counts in primary tumor and bone metastasis whole FOV images. (**D**) Macrophage phenotype counts in primary tumor and bone metastasis tumor tissue segments. (**E**) Macrophage phenotype counts in primary tumor and bone metastasis stromal tissue segments. (**F**) Kaplan-Meier curve of overall survival from mBC bone metastasis diagnosis to death in patient cohort based on high (*n* = 6) or low (*n* = 8) CD68^+^/pSMAD1-5-9^+^ macrophages in the tumor segment of mBC bone. Data are presented as mean ± SEM. Statistical values determined by Student’s *t* test (**B**–**E**). Statistical value determined by log-rank test (**F**).

To reconcile our findings of gene expression for myeloid activation unique to the bone TME, yet unchanged macrophage numbers between breast and bone locations, we next sought to examine more complex macrophage phenotypes. To distinguish activation states of macrophages, analysis of our 16 matched primary BC tumors and bone metastases was performed with a tailored Polaris mIHC panel (Figure 4A and Supplemental Table 9). The inflammatory and epigenetic markers C-Jun and H3K27ac were used as indicators of macrophage transcriptional and functional activation (21, 22). Additionally, the canonical BMP downstream signaling marker pSMAD1-5-9 was used to assess active BMP signaling. Interestingly, CD14^+^ myeloid cells, CD68^+^ macrophages, and immunosuppressive M2-like CD68^+^/CD163^+^ macrophages were not significantly distinguished between primary breast and bone TMEs (Figure 4B). Additionally, PanCK^+^ tumor cells and CD66b^+^ neutrophils were not distinguished between primary breast and bone TMEs (Supplemental Figure 4A). CD68^+^/CD163^+^ M2-like immunosuppressive macrophages were also not distinguished between primary breast and bone tumor and stromal tissue segments (Supplemental Figure 4, B and C). CD68^+^/C-Jun^+^ and CD68^+^/H3K27ac^+^ activated macrophages were also unchanged between the whole FOV images of primary tumors and bone metastases (Figure 4C). However, BMP signaling CD68^+^/pSMAD1-5-9^+^ macrophages had a trending enrichment in bone metastases compared with primary tumors (Figure 4C). Yet in the spatially restricted compartments of the tumor and stromal tissue segments, macrophages with activation markers and BMP signaling were unchanged in the primary and bone tissues (Figure 4, D and E).

To examine whether macrophage BMP signaling in the mBC bone TME affects patient survival, we performed survival analysis of patients with mBC who exhibited high versus low abundance of CD68^+^/pSMAD1-5-9^+^ macrophages in the tumor segments of mBC bone lesions. The limited sample size of our clinical cohort resulted in no statistically significant difference in overall survival from bone metastasis diagnosis in our patient cohort (Figure 4F). However, patients with high macrophage BMP signaling in the tumor tissue segment of bone samples exhibited an emerging trend of poorer overall survival compared with patients with low macrophage BMP signaling. Three years after bone metastasis diagnosis, patients with high macrophage BMP signaling in tumor segments of metastatic bone (*n* = 6) exhibited a 50% probability of survival compared with 80% for patients with low macrophage BMP signaling (*n* = 8) (Figure 4F). These results indicate that macrophage activation phenotypes can persist in both the primary tumor site and bone metastases, but BMP signaling in macrophages is distinct to the bone TME.

### BMP inhibition modulates models of metastatic BC in bone.

Due to the discovery of elevated macrophage BMP signaling in mBC bone metastases, the effect of BMP inhibition on macrophage activation was investigated. Macrophages, once differentiated from monocytes, can be activated with distinct bacterial products that entrain tumor suppressive functions. LPS is a common treatment to polarize or activate macrophages to mount antitumor effects (23). Another bacterial product, β-glucan, has also been used as a myeloid activation treatment to drive sustained myeloid memory proinflammatory and antitumorigenic functions through innate immunity (7). To examine the activation or entrainment of macrophages dependent on myeloid memory and BMP signaling, inflammatory responses in blood and bone marrow were examined in C57BL/6 (B6) mice. Mice were treated with β*-*glucan 7 days before receiving a secondary stimulus of LPS. The next day, intracardiac blood and bone marrow were collected for gene expression analysis of the inflammatory cytokines *Il-6* and *Tnfa*, which are markers of myeloid memory. In the blood, combined β*-*glucan and LPS trained immunity treatment significantly increased *Tnfa* gene expression (Supplemental Figure 5A). However, no significant changes in *Il-6* gene expression were observed in the blood, and no significant changes in *Tnfa* and *Il-6* were measured in the bone marrow upon trained immunity treatment (Supplemental Figure 5, B–D).

**Figure 5:**
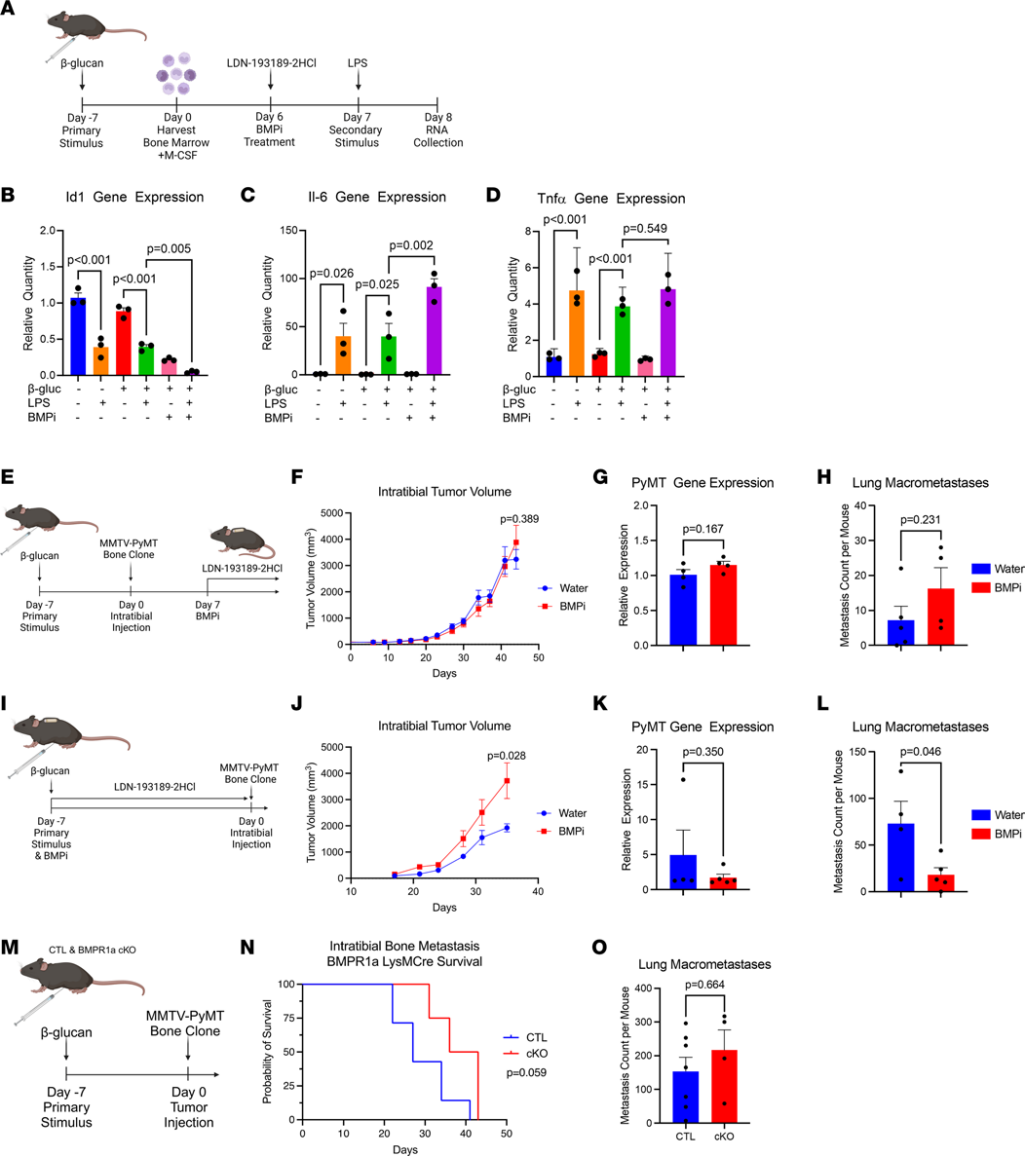
BMP inhibition modulates a model of metastatic breast cancer in bone. (**A**) Experimental scheme of BMDM isolation and treatment from mice stimulated with β-glucan. BMDM were treated with BMP inhibitor; after 24 hours, cells were stimulated with LPS for 24 hours. (**B**) BMDM *Id1* gene expression. (**C**) BMDM *Il-6* gene expression. (**D**) BMDM *Tnf* gene expression. (**E**) Experimental scheme for mouse model of bone metastasis with β-glucan stimulus and BMP inhibition (*n* = 5). Seven days prior to seeding a syngeneic bone metastasis cell line in the tibia, mice were stimulated with β-glucan. Twenty-eight–day osmotic pumps were implanted into mice 7 days after intratibial injection. (**F**) Intratibial tumor volume. (**G**) Peripheral blood *PyMT* gene expression. (**H**) Lung macrometastases. (**I**) Experimental scheme for mouse model of bone metastasis with β-glucan stimulus and BMP inhibition (*n* = 5). Seven days prior to seeding a bone metastasis cell line in the tibia, mice were stimulated with β-glucan, and 7-day osmotic pumps were implanted into mice. Intratibial injection of a syngeneic bone metastasis cell line was performed after 7 days. (**J**) Intratibial tumor volume. (**K**) Peripheral blood *PyMT* gene expression. (**L**) Lung macrometastases. (**M**) Experimental scheme for mouse model of bone metastasis with β-glucan stimulus in LysMCre CTL (*n* = 7) and LysMCre BMPR1a-cKO (*n* = 4) mice. Seven days prior to seeding a bone metastasis cell line in the tibia, mice were stimulated with β-glucan. (**N**) Kaplan-Meier curve of overall survival from intratibial bone metastasis seeding to mouse sacrifice or death. (**O**) Lung macrometastases. Data are presented as mean ± SEM. Statistical values determined by ordinary 1-way ANOVA followed by post hoc analysis for Tukey multiple comparisons (**B**–**D**). Statistical values determined by Student’s *t* test (**F**–**H**, **J**–**L**, and **O**). Statistical value determined by log-rank test (**N**).

To examine how BMP signaling modulation affects myeloid memory, trained immunity in bone marrow–derived macrophages (BMDM) was examined. B6 mice were treated with β*-*glucan 7 days prior to bone marrow collection and plating to differentiate into BMDM (Figure 5A). After 6 days in culture differentiating into macrophages, the BMDM were treated with the BMP inhibitor LDN-193189 2HCl, followed by LPS stimulus the next day. After 24 hours of acute LPS stimulus, RNA was collected for analysis of myeloid memory and BMP transcriptional control. Gene expression of the canonical BMP signaling gene *Id1* revealed a significant reduction in BMP signaling with LPS treatment (Figure 5B). *Id1* gene expression was also significantly decreased with BMP inhibitor treatment, as expected. Gene expression of the inflammatory cytokine *Il-6* showed increased gene expression with LPS stimulation (Figure 5C). However, treatment with β*-*glucan and LPS did not alter *Il-6* gene expression compared with the LPS treatment alone, suggesting that trained immunity stimulus did not affect *Il-6* cytokine production in BMDM. Interestingly, an even stronger induction of *Il-6* gene expression was observed with the combined treatments of β*-*glucan, LPS, and BMP inhibitor. *Tnfa* also exhibited a significant increase in gene expression with LPS stimulation (Figure 5D). Combined β*-*glucan and LPS treatment again did not increase *Tnfa* gene expression compared with the LPS-treated BMDM. Furthermore, BMP signaling inhibition in combination with β*-*glucan and LPS treatment did not enhance gene expression compared with LPS and β*-*glucan. For all 3 genes, β*-*glucan treatment in combination with LPS was not significantly altered compared with LPS alone; thus, LPS was sufficient to induce transcriptional activation of *Il-6* and *Tnfa* (Figure 5, C and D). This study suggests that, combined, BMP inhibition and macrophage activation stimuli increase proinflammatory *Il-6* production.

To examine whether macrophage activation affects mammary carcinoma progression in mouse models, B6 mice received a stimulus of β*-*glucan 7 days prior to seeding of syngeneic B6 MMTV-PyMT bone passaged mammary carcinoma cells (Supplemental Figure 6A). The β*-*glucan treatment did not alter the growth of orthotopic mammary fat pad tumors in syngeneic mice (Supplemental Figure 6B). Similarly, intratibial bone metastases did not display altered tumor outgrowth with β*-*glucan treatment (Supplemental Figure 7, A and B). This bone metastasis model allowed for reflection of the large number of tumor cells observed in our clinical cohort of mBC bone metastases. Histopathology analysis of the bone metastases revealed no substantial changes in morphology, with discernible bone trabecula, cortical bone, and the growth plates in both treatment groups (Supplemental Figure 7C). Importantly, this model also allowed for metastases to travel from the bone to the lungs to measure metastatic progression. No differences were observed in circulating tumor cell *PyMT* gene expression in peripheral blood (Supplemental Figure 7D). Secondary metastatic seeding from the bone to the lungs was observed in both mouse groups, but no statistical difference in the number of lung macrometastases was observed (Supplemental Figure 7E).

To determine if BMP signaling inhibition in the bone compartment could overcome the restrictions on β*-*glucan trained immunity response observed in our mammary carcinoma mouse models, the BMP inhibitor LDN-193189 2HCl was investigated in bone metastasis mouse models. We sought to determine if BMP inhibition administered to β*-*glucan entrained mice bearing mammary carcinoma bone metastases could restrict tumor progression, circulating tumor cells, and secondary metastasis to the lungs. To test the effect of BMP inhibition on established bone metastases, systemic LDN-193189 2HCl treatment began 7 days after seeding bone metastases and lasted for 28 days; then, mice were sacrificed 9 days later (Figure 5E). BMP inhibition did not alter the growth of well-established existing bone metastases in the tibia (Figure 5F). Histopathology analysis of the bone metastases revealed no marked changes in morphology in water and BMP inhibitor–treated mice (Supplemental Figure 8A). To determine if the 28 days of systemic BMP inhibition resulted in altered BMP signaling in the bone TME on sacrifice at day 44, IHC staining for pSMAD1-5-8 was performed on the bone metastases. BMP signaling with nuclear pSMAD1-5-8 staining was observed in the intratibial bone metastases of mice treated with water and BMP inhibitor (Supplemental Figure 8B). Quantitation of pSMAD1-5-8 staining revealed no significant difference in BMP signaling between treatment groups 9 days after systemic BMP inhibition ended (Supplemental Figure 8C). BMP inhibition did not affect the amount of circulating tumor cells in the blood measured by *PyMT* gene expression (Figure 5G). Secondary metastasis from the bone to the lungs was not affected by BMP inhibition (Figure 5H).

Since BMP signaling plays an important role in differentiation in the bone marrow, the early inhibition of BMP signaling during trained immunity reprogramming was examined prior to bone metastasis seeding. BMP signaling was inhibited between β-glucan stimulus and bone metastasis seeding in mice with osmotic pump delivery of the BMP inhibitor for 7 days (Figure 5I). Interestingly, early BMP inhibition during trained immunity reprogramming resulted in increased tibia bone metastasis growth (Figure 5J). Histopathology analysis of the tibia bone metastases revealed no substantial changes in morphology in water and BMP inhibitor–treated mice (Supplemental Figure 9A). Extravasated circulating tumor cells in the blood were not affected by BMP inhibition during training (Figure 5K). However, secondary metastasis from the bone to the lungs was significantly reduced in mice treated with the BMP inhibitor (Figure 5L). To determine if macrophage infiltration into the bone and lung metastases was altered by BMP inhibition during myeloid memory reprogramming, IHC staining for F4/80 was performed on the intratibial bone metastases and cleared lungs. Macrophage infiltration was observed in the intratibial bone metastases of mice treated with water and BMP inhibitor during trained immunity reprogramming (Supplemental Figure 9B). Quantitation of F4/80 staining revealed no significant difference between treatment groups (Supplemental Figure 9C). F4/80 IHC of lung metastases also revealed macrophage infiltration in both water and BMP inhibitor treatment groups (Supplemental Figure 9D). Quantitation of F4/80 IHC staining displayed no difference in staining of lung metastases between treatment groups (Supplemental Figure 9E). These studies suggest that distinct timing windows of BMP inhibition regulate the reprogramming of myeloid cells in mBC bone metastases.

To examine how BMP signaling in myeloid cells affects myeloid memory and bone metastases in mice, a LysMCre BMPR1a transgenic mouse model was utilized. LysMCre control (CTL) and LysMCre myeloid cell–restricted BMPR1a–conditional KO (BMPR1a-cKO) mice were used to examine whether myeloid cell BMP signaling was necessary or sufficient alongside β-glucan trained immunity stimulus to alter mammary carcinoma bone metastasis progression. CTL and BMPR1a-cKO mice were i.p. injected with β-glucan 7 days prior to intratibial injection of the syngeneic MMTV-PyMT mammary carcinoma bone clone to seed bone metastases (Figure 5M). Survival analysis revealed that BMPR1a-cKO mice had a strong trend of extended survival compared with CTL mice (Figure 5N). These results indicate that loss of BMP signaling in myeloid cells can prolong survival of mice with bone metastases when treated with β*-*glucan. Histopathology analysis of the tibia bone metastases revealed no marked changes in morphology in CTL and BMPR1a-cKO mice (Supplemental Figure 10A). All mice displayed secondary metastasis to the lungs from their bone metastases, yet quantitation of lung macrometastases revealed no significant alterations between groups (Figure 5O).

## Discussion

The goal of this study was to identify the landscape of the mBC bone TME and to demonstrate how it can now be effectively used in the diagnosis of patient samples and therapeutic development. Molecular and cellular profiling of BC primary tumor tissue has been instrumental in the selection of treatment regiments to enhance clinical responses. Despite mBC most commonly metastasizing to the bone, biopsies or surgical resections of bone lesions are not universally performed (24). Biopsy of bone metastases presents a surgical challenge as well as a technical challenge, as decalcification processing for FFPE tissues results in poor-quality nucleic acids and proteins (25). Additionally, the collection of bone biopsies is selectively used to determine hormone receptor (HR) status at the metastatic site to guide endocrine therapy (26). Consequently, mBC bone samples are underutilized in diagnosis and precision oncology, restricting the implementation of the bone TME as well as the advancement of therapies that may benefit patients with mBC. Since clinical guidelines now recommend biopsy of potential bone metastatic lesions, the availability of bone samples for analysis and knowledge obtained from these tissues will dramatically advance the understanding of the mBC bone TME (27).

HR^+^ BC is often considered an immune desert, with poor immune cell infiltration, low antigen presentation, and limited response to immune checkpoint blockade (28). In this study, patients with a high myeloid compartment gene signature exhibited inflammatory gene expression as well as a trend of extended overall survival. This was surprising, as myeloid cells in the mBC bone TME have commonly promoted mBC bone lesions (29). Our cohort of mBC patient bone metastases demonstrated overall low immune checkpoint target gene expression. The only immune checkpoint genes highly expressed in this mBC bone cohort were *CD276* (B7-H3) and *VSIR* (VISTA), which are both targets of emerging immunotherapy strategies (30, 31). Our results corroborate findings that only 12% of mBC bone lesions are PD-L1^+^ (32). The regional spatial analysis of the TME of mBC bone lesions revealed heterogeneous proinflammatory and immunosuppressive myeloid cell functions in the stroma. These results support the convention that myeloid cells have diverse functions in the mBC TME (33).

Spatial omics analysis of clinical patient tissues with multiplexed platforms has greatly advanced the study of immune TMEs. These analytical techniques have allowed for identification not only of cellular subpopulations in the TME, but also coexpression patterns, intratumoral heterogeneity, tissue segmentation, and cell-to-cell distances (34). Our analysis of clinical mBC bone metastases with tailored mIHC panels revealed the bone TME as primarily immune excluded, with higher myeloid cell populations in the stroma than in the tumor, while T cells were present in both compartments equally. GeoMx DSP analysis of a HER2-enriched BC primary tumor cohort has also shown macrophages were more enriched in the stroma than the tumor tissue segments (35). Additionally, mIHC analysis of a triple-negative BC primary tumor cohort has demonstrated that tumor-infiltrating lymphocytes (TILs) were more abundant in the stroma than the tumor segments (36). Tumor-promoting and immunosuppressive macrophages were significantly enriched in the stroma of our mBC bone metastases. However, we found that proinflammatory CD68^+^/pSTAT3^+^ and CD68^+^/HLA-DR^+^ macrophages were present both in the tumor and stroma of bone metastases. Unique proinflammatory macrophage and immunosuppressive macrophage spatial neighborhoods have been described in samples from patients with lung cancer profiled by imaging mass cytometry (37). The localization of macrophages and their functional phenotypes in the tumor or stroma of the BC TME have been shown to affect clinical outcome. For instance, in primary basal-like BC samples, high expression of tumor-promoting macrophage infiltration in the stroma, but not in the tumor, was associating with poor clinical outcome (38). These findings reinforce the need for spatial understanding of the immune TME to identify markers for clinical diagnosis and therapeutic intervention.

Primary tumors from patients with BC have been more extensively studied than mBC bone lesions, and the comparison of these 2 unique TMEs can help determine the tumor cell–intrinsic and organ-specific drivers of the bone TME. Differential gene expression analysis from our study’s matched mBC patient primary tumor and bone metastasis cohort revealed enhanced inflammatory myeloid genes in bone samples. This was surprising, since the metastatic bone TME has been shown to be myeloid enriched but immunosuppressive in mouse models of mBC bone metastasis as well as in clinical samples (4, 39). However, macrophages derived from inflammatory monocytes in the bone have been shown to promote mBC bone metastases in both mice and humans (40). When we compared matched mBC patient primary tumors with the bone, we found significantly decreased total immune cells, T cells, and B cells in the bone. However, our myeloid cell, neutrophil, and macrophage populations remained unchanged in number. Our results were distinct from nonspatial publicly available bulk gene expression data sets, which have revealed lower macrophage populations in bone metastases compared with primary tumors, while neutrophils were higher in bone metastases (41). However, the bone is a primary reservoir for myeloid cells, which can exhibit proinflammatory or immunosuppressive functions in health and disease (3). Thus, functional investigation of myeloid phenotypes in the bone with spatial contexts can provide a deeper understanding of the pathogenesis of bone metastases. Macrophage activation states in the TME are extensive beyond the bifurcated M1-like tumor suppressive and the M2-like tumor-promoting macrophages (5). We found that C-Jun and H3K27ac, markers of inflammatory transcriptional and epigenetic activation, were present in macrophages in the TME of both primary tumors and bone metastases. These proteins are important markers of memory reprogramming for trained immunity in myeloid cells as well as epithelial cells (42, 43).

BMP signaling plays an important role in promoting and suppressing cancer and is currently being investigated as a therapeutic target to modulate both tumor cells and the cells in the TME (44). Gene expression database analysis has shown that individual BMP family members correlate with improved or diminished survival of patients with BC, demonstrating the context-dependent nature of BMPs in BC (45). Our study identified *BMP2* as the top significantly enriched gene in mBC patient bone metastases compared with primary BC tumors. BMP family genes were significantly enriched in gene expression databases of mBC in bone (46). However, the origin of BMP ligands in the bone TME remains unclear, as macrophages, tumor associated macrophages, and BC cells have all been shown to secrete BMP2 (18–20). In our patient cohort, CD68^+^/pSMAD1-5-9^+^ macrophages with BMP signaling were increased in the bone compared with the primary tumor. Importantly, patients with high macrophage BMP signaling had a trend of poorer overall survival. High expression of the BMP receptors BMPR1a and BMPR2 in BC correlates with significantly worse patient survival (14, 47). Our findings of both BMP2 enrichment in mBC bone lesions as well as BMP signaling macrophages with pSMAD1-5-9 expression supported our investigation into the function of myeloid cell BMP signaling in bone metastasis. Our previous work revealed that BMP signaling inhibition in macrophages results in a more proinflammatory and tumor suppressive phenotype (13). BMPs promote osteoblast differentiation and activation to drive osteogenesis (47). However, BMPs have also been shown to promote the vicious cycle of osteolytic tumor–induced bone disease by supporting osteoclast bone resorption and regulating osteoblast-osteoclast crosstalk (16). Taken together, the elevated BMP signaling we observed in the bone may be an important therapeutic diagnostic or treatment opportunity for bone metastases and associated tumor-induced bone disease.

Trained immunity is currently being investigated as a cancer therapeutic to drive macrophage reprogramming in the TME (6). Multiple mouse models have established that myeloid cells in the TME can undergo reprogramming to be antitumorigenic due to trained immunity stimulus (7, 48). Myeloid cells have been shown to infiltrate into the tumors of mouse tumor models and exhibit antitumorigenic functions to restrict tumor growth and prolong mouse survival after induction of trained immunity with β-glucan treatment (7, 8). Trained immunity stimulus results in lung interstitial macrophage reprogramming, which significantly reduced lung metastasis and prolonged survival in multiple mouse models (48). A trained immunity–inducing nanobiologic has also established antitumorigenic myeloid reprogramming in the bone marrow to restrict tumor progression (8). Since trained immunity reprogramming occurs in the myeloid progenitor cells of the bone marrow and the bone marrow is a distinct mBC niche, we aimed to examine the effects of trained immunity stimuli in mouse models of mBC bone lesions (9).

Our analysis of trained immunity responses revealed distinct niche-dependent phenotypes in mice. We found that bone marrow did not exhibit changes to *Il-6* and *Tnfa* gene expression with trained immunity stimuli. However, a systemic response to trained immunity was observed in the blood, with increased *Tnfa* gene expression after mice received trained immunity stimulus. In BMDM, cytokine gene expression was unchanged with trained immunity treatment or BMP inhibitor treatment alone. We found that BMDM treated with a BMP inhibitor in combination with β-glucan and LPS had increased proinflammatory *Il-6* gene expression. Recombinant BMP-7 treatment has been shown to decrease macrophage IL-6 production (49). This modulation of IL-6 expression indicates that the high BMP signaling in the bone niche has the capacity to both suppress and enhance macrophage activation states. These results suggests that the bone marrow is a unique environment that may exhibit distinct challenges to elicit myeloid memory due to high BMP signaling.

The results of combined BMP signaling modulation and trained immunity stimulus in our mouse models of mBC bone metastasis demonstrate that the timing of these stimuli affect metastatic progression. Systemic LDN-193189 treatment has been shown to promote bone metastasis formation in immune-deficient mice (50). Systemic BMP inhibition has also been shown to restrict tumor growth in multiple mouse models of cancer, including ovarian, colorectal, lung, prostate, and BC (13, 51–54). In an immune-competent mammary carcinoma spontaneous metastasis model, systemic BMP inhibition resulted in a reduced primary tumor burden as well as decreased secondary metastasis to the lungs (14). Importantly, this study showed a restriction of tumor-promoting macrophage phenotypes. Conversely, studies have demonstrated that BMP4, which also signals through BMPR1a, restricts myeloid-derived suppressor cell activity and the formation of mBC bone metastases in mice (55, 56). Our study’s results revealed that combined trained immunity and BMP signaling inhibition, but not β-glucan alone, was sufficient to restrict the progression of an aggressive mouse model of mBC intratibial bone metastasis. These results reflect the complex roles of BMP signaling in the TME (57).

Our study provides a strong rationale for further investigation of targeting macrophage activation states in mBC bone lesions. Future studies in mouse models of mBC bone lesions could differentiate the role of BMPs through distinct BMP ligand and receptor signaling functions. Investigations into the timing of BMP signaling during the treatment of primary BC tumors, mBC micrometatases, or established bone lesions would help establish the translational potential of BMP modulation for patients with mBC. Additional targets from our bulk gene expression analysis of the bone TME could be investigated for combinatorial therapeutic approaches with BMP inhibition. The monocyte and macrophage chemokine receptor *CCR2* and chemokine *CCL4* were enriched in mBC bone samples, and BMP signaling has been shown to promote monocyte chemotaxis, adhesion, and macrophage differentiation (58). BMP signaling inhibition has been shown to reduce PD-L1 expression on DCs to improve T cell activation, and BMP inhibition sensitizes tumors to anti–PD-1 immunotherapy in mice (59, 60). mBC bone lesions have exhibited poor response to immunotherapy, supported by our findings of low *CD274* (PD-L1) and *CD279* (PD-1) expression in mBC bone tissue. However, we discovered high gene expression of *CD276* (B7-H3) and *VSIR* (VISTA) in our bone metastasis samples. These findings suggest that other BMP inhibitor therapeutic combinations could be studied in the context of bone metastases, including macrophage chemotaxis and immune checkpoint inhibitors.

The advancement of bone metastatic tissue omics analysis has set the stage for future discoveries of unique cellular and signaling signatures in the bone TME. This study distinguished macrophage activation states in mBC primary tumor and bone tissues, with macrophage BMP signaling distinct to the TME of bone metastases. In order to develop therapies that reprogram the myeloid TME, defining the function of BMP signaling in the bone must be fully understood.

## Methods

### Histology and IHC.

After mouse sacrifice and necropsy, harvested mouse tissues underwent FFPE processing. All tissues were fixed in 10% neutral buffered formalin for 24 hours. Soft tissues were then placed in 70% ethanol. Bone tissues were decalcified in 14% EDTA (pH 7.6) for 5–10 days prior to transfer to 70% ethanol. Then the University of Colorado Cancer Center Pathology Shared Resource embedded the mouse tissues in paraffin, and they were then sectioned at 5 μm thickness and mounted on positively charged microscope slides. Deidentified archival FFPE human clinical tissue samples from BC primary tumors and mBC bone metastases were also sectioned at 5 μm thickness and were then mounted on positively charged microscope slides. All slides were baked for 1 hour at 60°C.

Mouse and human FFPE tissue slides were stained with H&E and IHC by the University of Colorado Cancer Center Pathology Shared Resource for histological analysis. H&E staining was performed with the Tissue-Tek Prisma autostainer platform (Sakura) with Gills No. 2 hematoxylin and 1% alcoholic eosin. IHC staining was performed with the Discovery Ultra autostainer platform (Roche). Primary antibodies for human CD3 (Dako, A425) at 1:200, human CD68 (Dako, MO814) at 1:750, pSMAD1-5-8-9 (MilliporeSigma, AB3848-1) at 1:50, and mouse F4/80 (Cell Signaling Technology, 70076) at 1:200 were used. Signal was detected by OmniMap polymer secondaries (Roche) to appropriate host and ChromoMap DAB chromogen substrate (Roche). All bright-field IHC and H&E slides were scanned at 40× (0.22 μm/pixel) magnification using an Aperio ScanScope XT System (Leica) for digital slide viewing with Aperio ImageScope (Leica). IHC quantitation was performed as previously described (13).

### Human gene expression panel.

Bulk gene expression analysis of archival patient primary tumors and bone metastases was performed on 20 μm sectioned FFPE scrolls. One to two 20 μm–thick scrolls were collected in RNase free tubes; then, RNA was isolated using the RNeasy FFPE Kit (Qiagen, 73504). nCounter PanCancer IO 360 panels (NanoString, XT-CSO-HIO 360-12) were utilized to measure the gene expression of mBC bone metastases and matched primary tumors. In total, 300 ng of input total RNA in 8 μL was utilized for the hybridization reaction, which ran for 24 hours. Hybridized samples were then loaded into nCounter SPRINT cartridges (NanoString) and run on the nCounter Sprint profiler (NanoString). Data were then analyzed using the nSolver Advanced Analysis software (NanoString v4.0).

### DSP.

BC patient bone metastasis regional protein expression was measured using the GeoMx Digital Spatial Profiler platform (NanoString). Clinical sample slides were sent to NanoString as part of the Technology Access Program (TAP), where slide staining and data acquisition was performed. ROIs were selected on FFPE sample slides by fluorescence staining with DAPI and PanCK, CD3, and CD68 antibodies. ROIs were selected for enrichment of PanCK, CD68, and CD3 staining. For each patient slide, 4 ROIs for PanCK-, CD68-, and CD3-enriched regions were selected with uniform diameters — 600 μm for PanCK ROIs and 300 μm for CD68 and CD3 ROIs. Raw counts from barcoded oligo probes derived from antibodies were normalized with internal spike-in controls to account for system variation.

### mIHC.

The Polaris mIHC platform (Akoya) was used to measure spatial single-cell protein expression with 9-color antibody panels in mBC patient primary tumors and bone metastases. Slide staining and imaging was performed by the Human Immune Monitoring Shared Resource at the University of Colorado Cancer Center. Multispectral image FOVs were selected with Phenochart (Akoya v1.0.9), with up to 20 regions selected per tissue. FOVs were selected for tumor and stroma interaction while avoiding adjacent bone and bone marrow. Image analysis was performed with InForm (Akoya v2.6.0) to spectrally unmix images, segment tissue, segment cell compartments, and phenotype cells. Nearest-neighbor spatial analysis and complex phenotype evaluation was then performed using phenoprReports (Akoya). For each patient, the average cell phenotype count was calculated.

### Cell line generation and cell culture.

The C57BL/6J MMTV-PyMT mammary carcinoma cell line was obtained from the laboratory of Harold Moses at Vanderbilt University, Nashville, Tennessee, USA. The B6 MMTV-PyMT cell line was cultured in DMEM high glucose with sodium pyruvate (Corning, 10-013-CV), 10% FBS (Thermo Fisher Scientific, 26140-079), and 1% Antibiotic-Antimycotic (Thermo Fisher Scientific, 15-240-096). To generate a bone metastasis clone of the B6 MMTV-PyMT cell line, 1 × 10^5^ B6 MMTV-PyMT cells were injected into the caudal artery of a syngeneic C57BL/6J female mouse to seed bone metastases (61). Once bone metastases were palpable in the tibia and femur, the tumor cells were collected from the bone and cultured in medium with10 μg/mL ciprofloxacin hydrochloride (Bioworld, 40310032). Cells were then passaged again through the bone by caudal artery injection. After 3 passages though the bone, the B6 MMTV-PyMT bone clone was expanded for experimental use. The cell line was routinely tested for mycoplasma infection by PCR, authenticated by morphology, and not cultured past passage 30.

### Mouse strains.

All animal work was performed at the University of Colorado Anschutz Medical Campus and housed under specific pathogen–free conditions. C57BL/6J (The Jackson Laboratory, 000664) female mice between 6 and 8 weeks of age were used for all studies. Transgenic BMPR1a LysMCre mice were bred onto a CD57BL/6J background and contained the following genotypes: LysMCre (B6.129P2-Lyz2^tm1[cre]Ifo^) (The Jackson Laboratory, 004781), mTmG Cre reporter (B6.129[Cg]-Gt[ROSA]26Sor^tm4[ACTB-tdTomato,-EGFP]Luo/J^) (The Jackson Laboratory, 007676), and BMPR1a cKO (B6;129S7-Bmpr1a^tm1Bhr^/Mmnc) (Mutant Mouse Resource and Research Centers, 016131-UNC). LysMCreBMPR1a^wt/wt^ROSA26^mTmG^ (CTL) or LysMCreBMPR1a^fl/fl^ROSA26^mTmG^ (BMPR1a cKO) mice were used for the transgenic mouse models.

### Mouse models of mammary carcinoma.

To generate intratibial bone metastasis tumors, mice were s.c. injected with 1 mg/kg of the analgesic buprenexSR by the University of Colorado Anschutz Veterinarian Technicians. The mice were anesthetized under isoflurane for the procedure. Tibias were injected using 27-gauge needles (Easy Touch, 827555) containing 1 × 10^5^ B6 MMTV-PyMT Bone Clone cells in 20 μL PBS or PBS alone as a sham control in the other tibia. To generate mammary fat pad tumors, mice were anesthetized under isoflurane and were then injected with 1 × 10^5^ B6 MMTV-PyMT Bone Clone cells in 100 μL PBS into the fourth mammary fat pad on both sides of the mouse using 27-gauge needles.

Mice were monitored by weekly weight measurement, observation, and palpation. Once tumors were palpable, measurements of tumor length and width were collected every 3–5 days with calipers, and animals were sacrificed after tumors reached the maximum measurement of 2.5 cm in any direction. Tumor volume was calculated by volume = (length × width^2^)/2. Peripheral blood from the heart was collected in RNAlater after sacrifice for later blood RNA isolation using the Mouse RiboPure Blood RNA Isolation Kit (Invitrogen, AM1951). Tissues including heparin-cleared lungs, spleens, and tibias were collected for histological analysis.

### Mouse models of trained immunity.

Mice were i.p. injected with 1 mg of β-glucan (Invivogen, tlr1-bpg) in 200 μL PBS or PBS alone as a vehicle control. After 7 days, mice were injected with B6 MMTV-PyMT Bone clone cells for mammary fat pad and intratibial tumor studies, injected i.p. with 35 μg LPS (Invivogen, tlr1-eblps) in 100 μL PBS for 24 hours before sacrifice, or mice were sacrificed for isolation of BMDM. After sacrifice, peripheral blood from the heart was collected in RNAlater for later blood RNA isolation using the Mouse RiboPure Blood RNA Isolation Kit (Invitrogen, AM1951). Bone marrow was flushed from the tibias with cold PBS and resuspended into single cells; then, RNA was isolated using the RNeasy Plus Mini Kit (Qiagen, 74134).

### BMDM trained immunity.

Seven days before harvesting bone marrow, female mice were treated with β-glucan or PBS control. BMDM were isolated using a protocol adapted from previously published methods (62, 63). Briefly, bone marrow was flushed from tibia and femur of mice using a 27-gauge needle syringe and DMEM high glucose with sodium pyruvate medium. The bone marrow from each treatment group was pooled and then centrifuged at 200*g* for 5 minutes at 4°C. Bone marrow was then resuspended in bone marrow culture medium containing DMEM high glucose with sodium pyruvate medium, 10% FBS, 1% Triple Antibiotics (Thermo Fisher Scientific), and 1 ng/mL mouse M-CSF1 with carrier (R&D, 416-ML-010). Bone marrow was then plated at 2 × 10^6^ cells in 2 mL bone marrow culture medium per well in 6-well plates. A total of 1 mL fresh bone marrow culture medium was added on day 4. BMDM were washed with PBS on day 6 and were then treated with 100 nM LDN-193189 2HCl (Selleck Chemicals, S7507) or water control in DMEM high glucose with sodium pyruvate medium containing 10% FBS and 1% Triple Antibiotics. On day 7, 10 ng/mL of LPS or PBS control was added to wells. Then on day 8, wells were washed with PBS and RNA was isolated using the RNeasy Plus Mini Kit.

### Mouse gene expression analysis.

RNA was purified using the RNeasy Plus Mini Kit or Mouse RiboPure Blood RNA Isolation Kit for gene expression analysis by quantitative PCR (qPCR) (Thermo Fisher Scientific). The iScript cDNA Synthesis Kit (Bio-Rad, 1708891) was used to generate cDNA. qPCR reactions were performed using the SsoAdvanced Universal SYBR Green Supermix (Bio-Rad, 172571) on a CFX qPCR instrument (Bio-Rad) or using the PowerUP Sybr Green (Thermo Fisher Scientific, A25742) on a QuantStudio 6 Flex instrument (Applied Biosystems). All genes were run in at least biological triplicate as well as technical triplicate or duplicate. GAPDH was used as the housekeeping gene to normalize gene expression.

### Mouse models of BMP inhibition.

The BMP inhibitor LDN-193189 2HCl was delivered systemically to mice at 3 mg/kg/day in osmotic pumps. Osmotic pumps continuously delivered LDN-193189 2HCl in water or water alone as a vehicle control. Osmotic pumps were implanted either on the same day as β-glucan treatment or 7 days after intratibial bone metastasis injection. Osmotic pumps delivered LDN-193189 2HCl for 7 days (Alzet, 0000290) or 28 days (Alzet, 0000298). To implant the osmotic pumps, mice were anesthetized under isoflurane for the procedure and then received 2 mg/kg bupivacaine (HF Acquisition) s.c. The pump was inserted into an incision in the skin above the scapula. The incision was then closed with wound-closing autoclips (Thermo Fisher Scientific, 22-275998), and mice received i.p. injections of 5 mg/kg Rimadyl (Zoetis) analgesic for 3 days.

### Statistics.

All experiments were performed in technical triplicate or duplicate and in biological triplicate. Raw data were recorded in Excel (Microsoft v16.67) and were then imported into Prism (GraphPad v9.4.1) for statistical analysis. Bar graphs demonstrate the mean, with individual data points visible and data reported by the mean ± SEM. Statistical analysis between 2 groups was performed with the Student’s *t* test (parametric, unpaired, 2-tailed) or the Mann-Whitney *U* test (nonparametric, unpaired, 2-tailed) when the data set was not normally distributed. Statistical analysis between more than 2 groups was performed with an ordinary 1-way ANOVA (parametric, unpaired) followed by post hoc analysis for Tukey multiple comparisons. Tumor growth curves were analyzed with a Student’s *t* test (parametric, unpaired, 2-tailed). Survival studies used a log-rank test for statistical significance. Correlation analyses used a Pearson correlation coefficient after log transformation of the data to adjust for outliers. The statistical analysis used in experiments can be found in the corresponding figure legend. All statistical tests used a cutoff *P* value of 0.05 for significance. To achieve an optimal power of 0.80 for mouse tumor metastasis and size differences, 5 mice per experimental group were used.

### Study approval.

The Pathology Shared Resource in the Department of Pathology at the University of Colorado Anschutz Medical Center has a Colorado Multiple-IRB–approved protocol (COMIRB 15-1641) to store tissue from clinical procedures. The specimens for this study came from remnant archival tissue from a surgical procedure. This research is considered nonhuman-subject research, since the material was provided in a deidentified manner to the study with the Pathology Shared Resource acting as the honest broker. Retrospective collection of demographic and clinical data from electronic medical records (UCHealth Epic system) was performed by KCT, who was blinded to experimental analysis results. Collection of these data are allowed for specimens contained in the Department of Pathology surgical specimen archive under COMIRB protocol 15-1641. Only deidentified data linked to the specimens by a study ID were supplied to the researchers. Clinical outcomes including overall survival were analyzed by number of days from bone sample collection to the date of last follow up or death. All experiments were randomized and blinded when possible. Mouse studies were in compliance with the *Guide for the Care and Use of Laboratory Animals* (National Academies Press, 2011) (protocol no. 00533).

### Data availability.

The underlying data will be made available by the corresponding author upon request. All deidentified or anonymized human subject data will be shared upon request when allowable. Values for all data points in graphs are reported in the Supporting Data Values file.

## Author contributions

CLI and PO performed study conceptualization and funding acquisition. Study methodology and investigation was performed by CLI, DMS, KLZ, KCT, EES, and JAC. Data visualization was performed by CLI, KCT, and JAC. CLI wrote the manuscript. Manuscript review and editing were performed by CLI, KCT, DMS, and PO. Project administration and supervision was provided by PO.

## Supplementary Material

Supplemental data

Supplemental tables 1-9

Supporting data values

## Version 1 01/09/2024

Electronic publication
