# Supplementary material for: Unique macrophage phenotypes activated by BMP signaling in breast cancer bone metastases
Source: JCI Insight. 2024 Jan 9;9(1):e168517. doi: 10.1172/jci.insight.168517 (PMC10906463; doi:10.1172/jci.insight.168517)
Supplement: Supplemental data [file jciinsight-9-168517-s119.pdf]

List of Supplementary Materials

Supplemental Figures 1 to 10

Supplemental Tables 1 to 9 (Excel file)

Supplementary Figures:

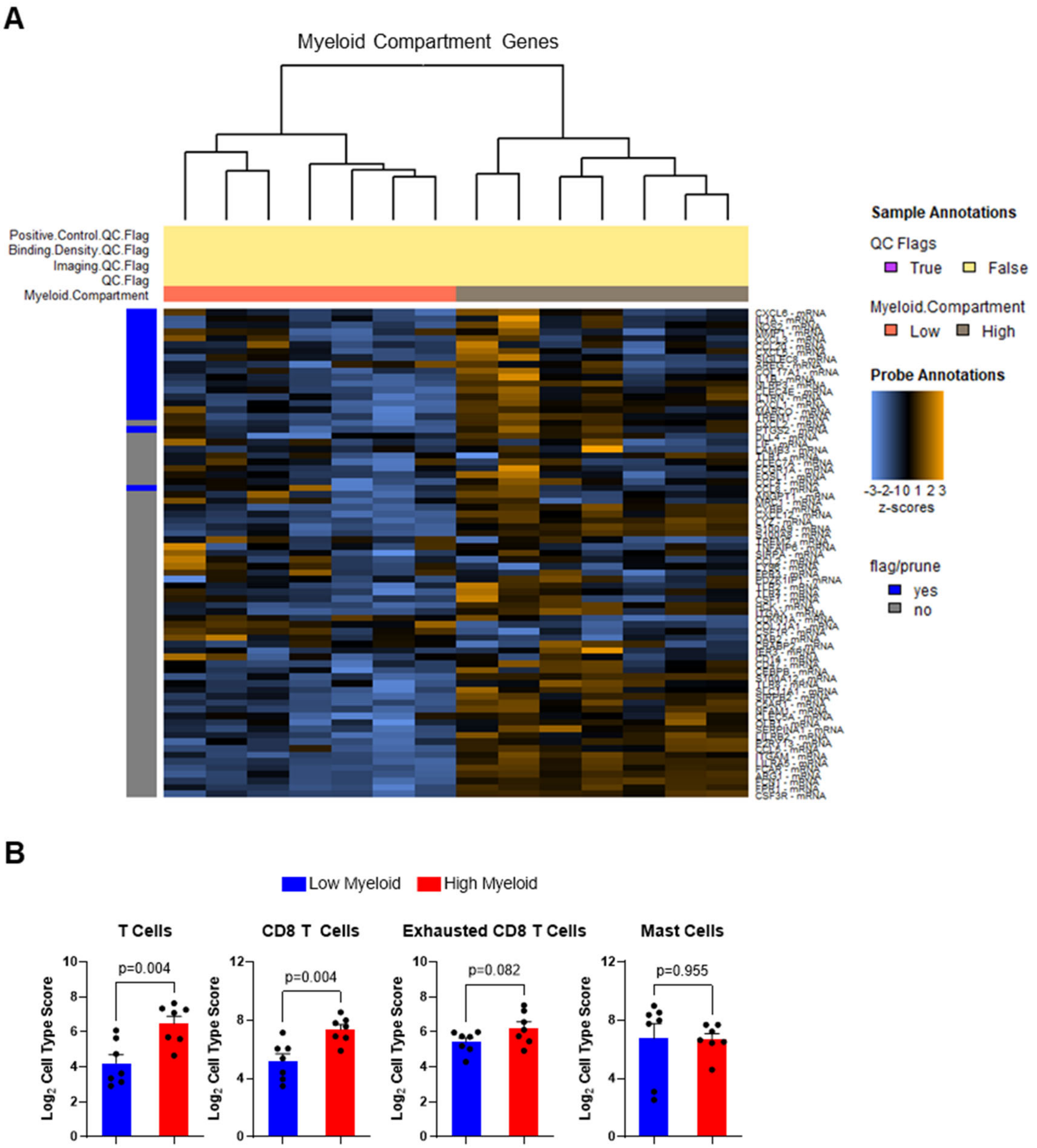

Supplemental Figure 1. **Differential Gene Expression of Breast Cancer Bone Metastases.** (**A**) Myeloid compartment genes from PanCancer IO 360 gene expression analysis of archival FFPE mBC patient bone metastases (n=14). Cohort clustered into samples exhibiting high myeloid compartment gene expression (n=7) and samples exhibiting low myeloid compartment gene expression (n=7). (**B**) Cell type gene signature scores for low myeloid compartment and high myeloid compartment patient samples. Data are presented as mean ± SEM. Statistical values determined by student’s t test (**B**).

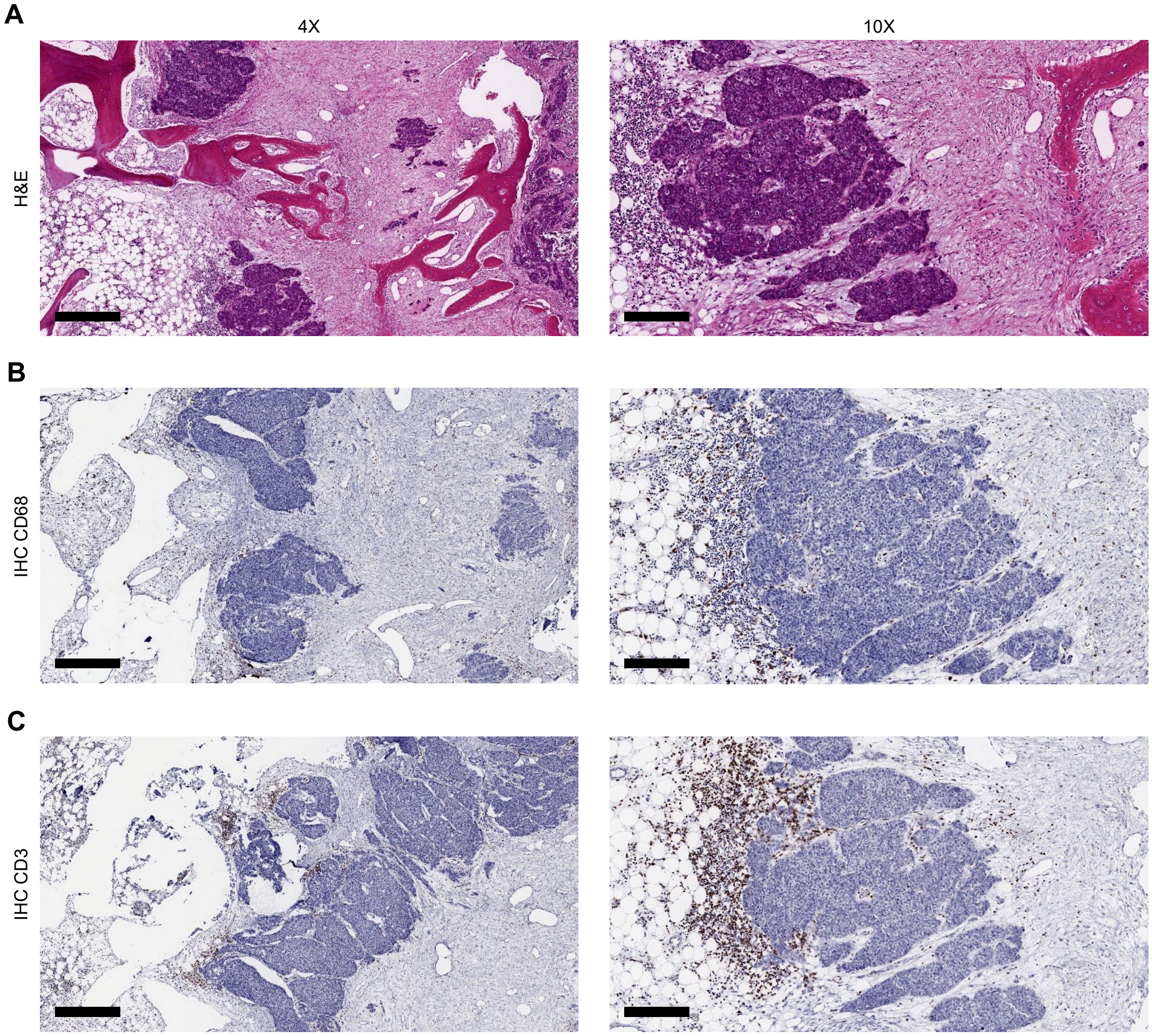

Supplemental Figure 2. Metastatic Breast Cancer in Bone Exhibits Immune Infiltration. (**A**) Representative images of mBC patient bone metastases with H&E staining. (**B**) Representative images of mBC patient bone metastases with CD68 IHC staining. (**C**) Representative images of mBC patient bone metastases with CD3 IHC staining. Images captured at 4X (left) and 10X (right) (**A-C**). Scale bar 500µm for 4X and 200µm for 10X (**A-C**).

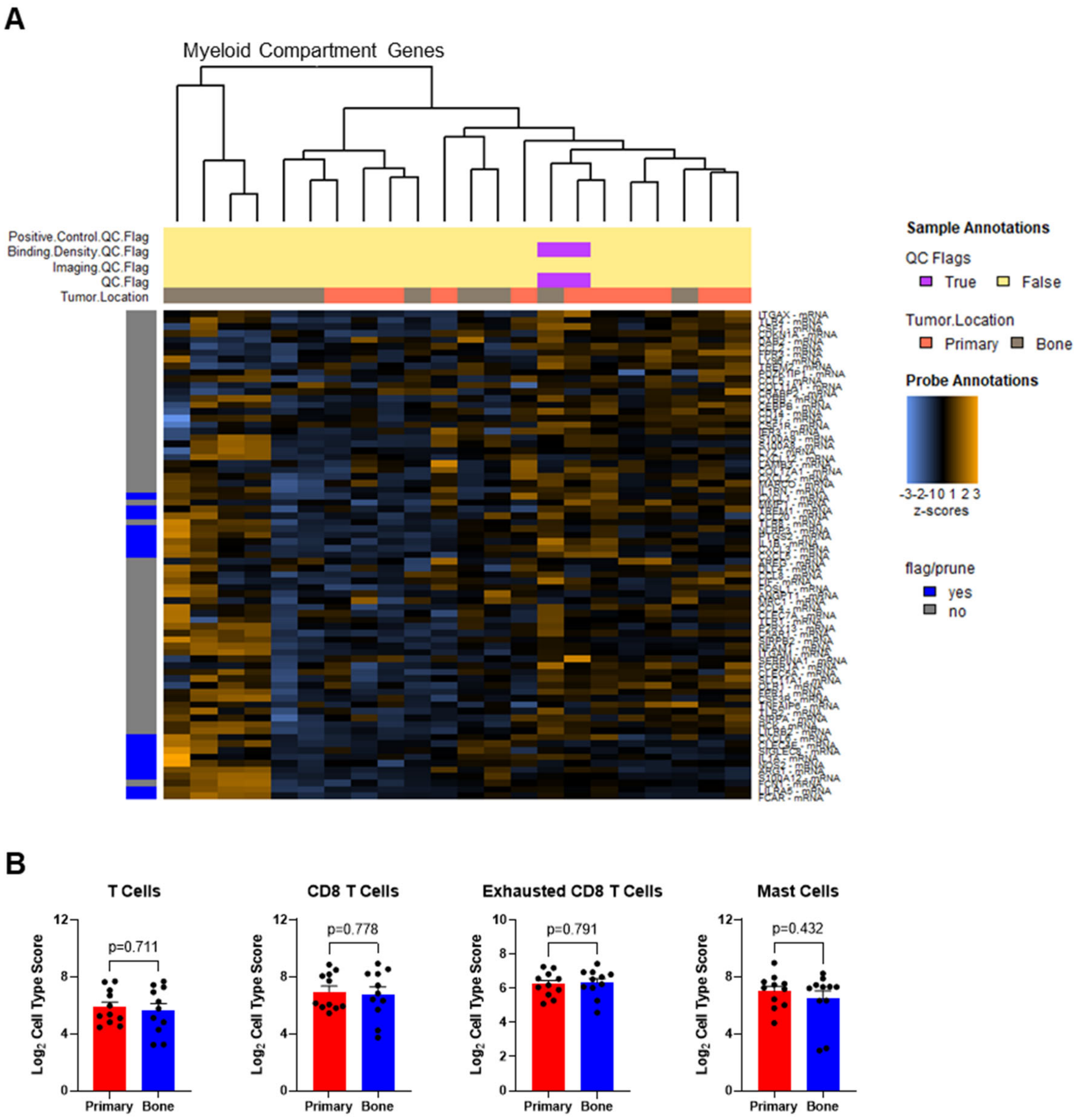

Supplemental Figure 3. Differential Gene Expression of Matched Breast Cancer Primary Tumors and Bone Metastases. (**A**) Myeloid compartment genes from PanCancer IO 360 gene expression analysis of archival FFPE mBC patient matched primary tumors and bone metastases (n=11). (**B**) Cell type gene signature scores for primary tumors and bone lesions. Data are presented as mean ± SEM. Statistical values determined by student's t test (**B**).

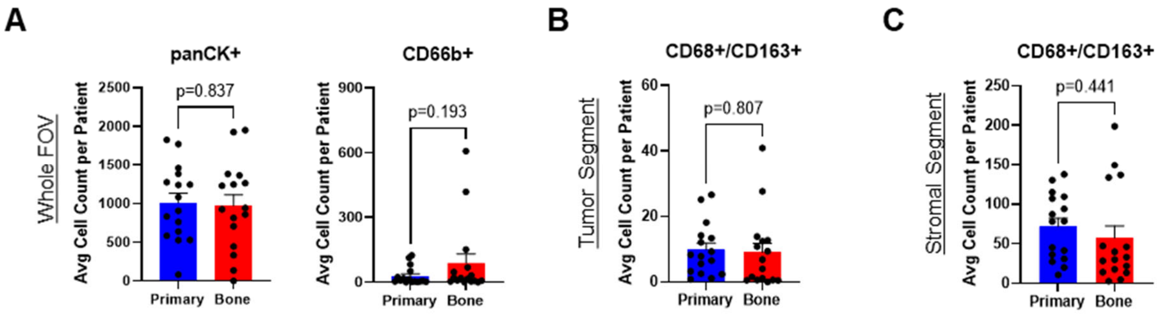

Supplemental Figure 4. Cell Phenotypes in Matched Metastatic Breast Cancer Primary Tumors and Bone Metastases. (**A**) Cell phenotype counts in primary tumor and bone metastasis whole FOVs from Polaris mIHC analysis of patient primary tumor and matched bone metastasis (n=16). (**B**) Macrophage phenotype counts in primary tumor and bone metastasis tumor tissue segments. (**C**) Macrophage phenotype counts in primary tumor and bone metastasis stromal tissue segments. Data are presented as mean ± SEM. Statistical values determined by student's t test (**A-C**).

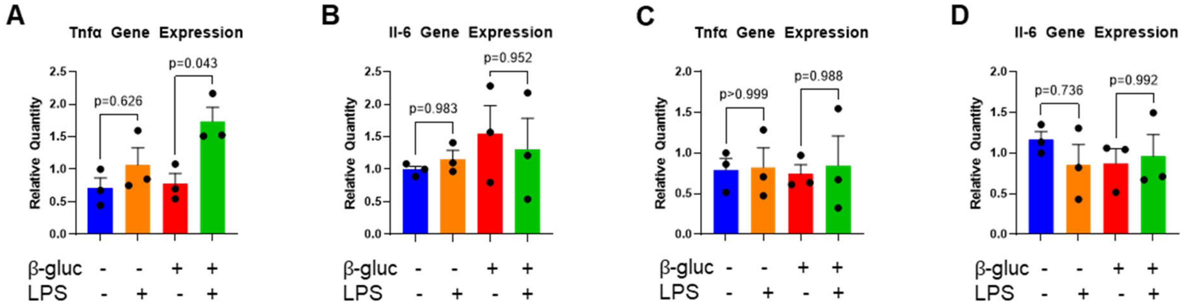

Supplemental Figure 5. Trained Immunity Stimulus Modulates Inflammatory Gene Expression. Mice were stimulated with β-glucan (n=6) or vehicle (n=6) 7 days before being stimulated with LPS (n=6) or vehicle (n=6) for 24hrs. Mice were then sacrificed, and RNA was isolated from blood and bone marrow for gene expression analysis by qPCR. (**A**) Peripheral blood *Tnfα* gene expression. (**B**) Peripheral blood *Il-6* gene expression. (**C**) Bone marrow *Tnfα* gene expression **(D)** Bone marrow *Il-6* gene expression. Data are presented as mean ± SEM. Statistical values determined by ordinary one-way ANOVA followed by post-hoc analysis for Tukey multiple comparisons (**A-D**).

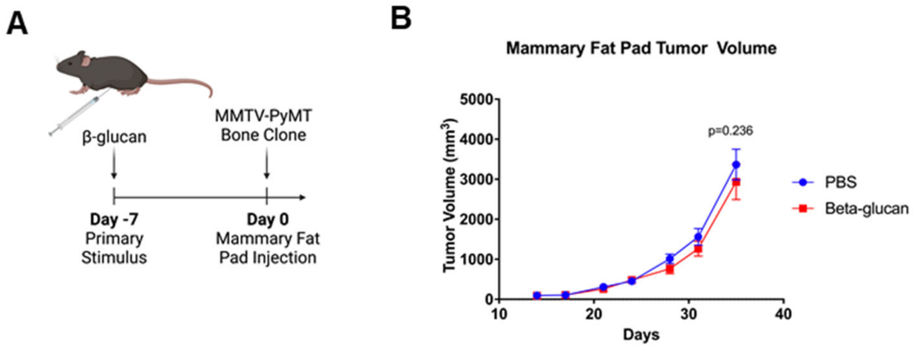

Supplemental Figure 6. Macrophage Activation in an Orthotopic Model of Mammary Carcinoma. (**A**) Experimental scheme for mouse model of mammary fat pad tumors with β-glucan stimulus (n=5). 7 days prior to seeding a syngeneic bone metastasis cell line in the mammary fat pad, mice were stimulated with β-glucan or vehicle. (**B**) Mammary fat pad tumor volume. Data are presented as mean ± SEM. Statistical values determined by student’s t test (**B**).

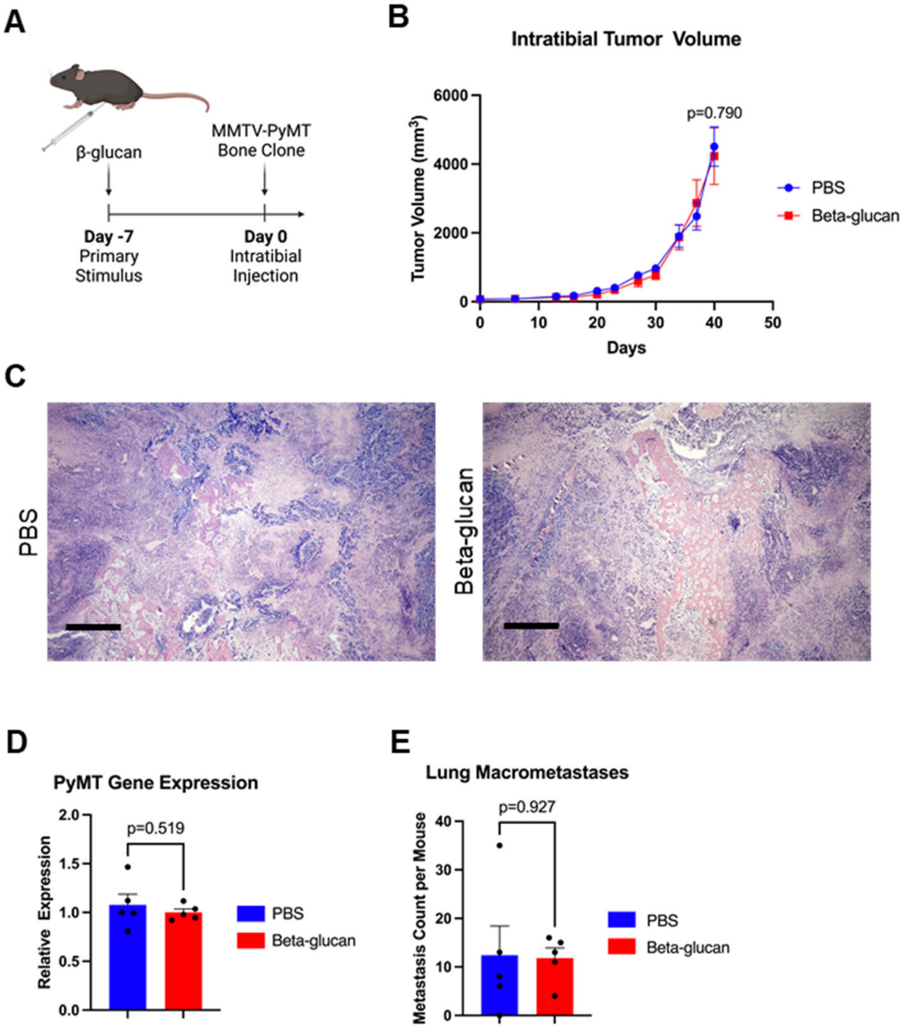

Supplemental Figure 7. Macrophage Activation in a Model of Mammary Carcinoma Bone Metastasis. (**A**) Experimental scheme for mouse model of mammary fat pad tumors and bone metastasis with β-glucan stimulus (n=5). 7 days prior to seeding a syngeneic bone metastasis cell line in tibia, mice were stimulated with β-glucan. (**B**) Intratibial tumor volume. (**C**) Representative image of H& E staining of intratibial tumor from PBS and β-glucan treated mice. Scale bars indicate 500µm for representative images. (**D**) Peripheral blood *PyMT* gene expression. (**E**) Lung macrometastases. Data are presented as mean ± SEM. Statistical values determined by student's t test (**B, D-E**).

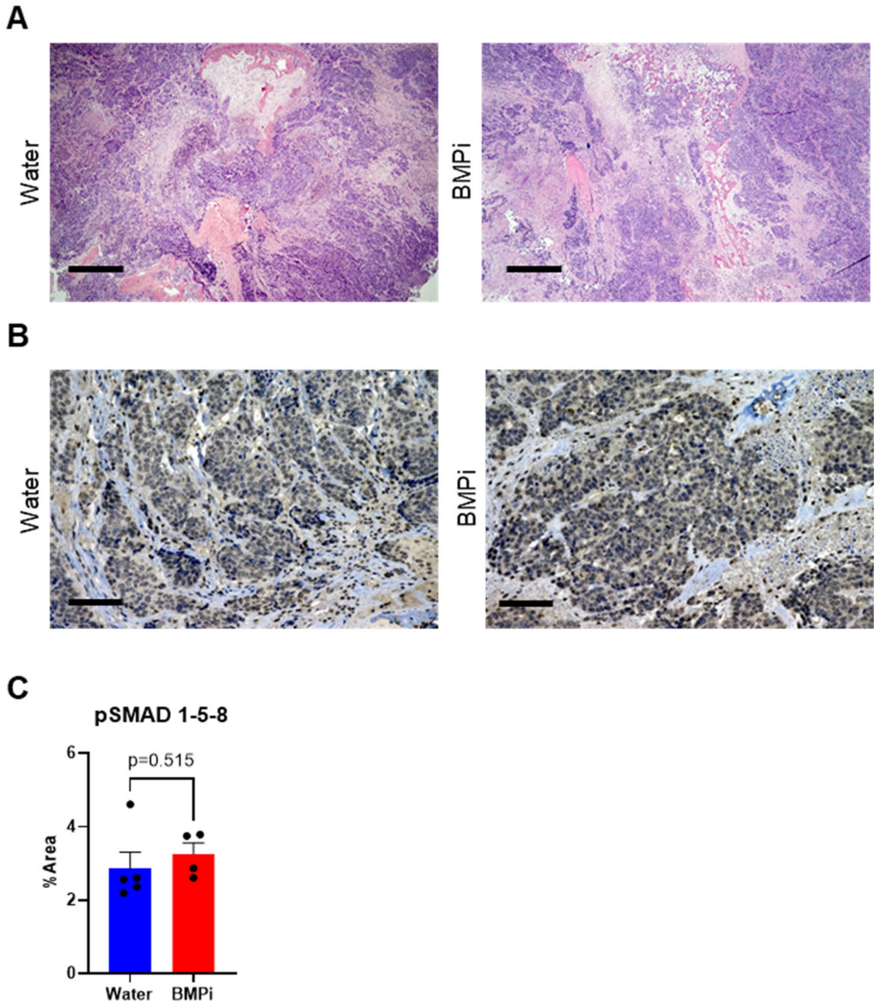

Supplemental Figure 8. Mammary Carcinoma Bone Metastases with BMP Inhibition. (**A**) Representative image of H&E staining of intratibial tumor from water and BMP inhibitor treated mice. Scale bars indicate 500µm for representative images. (**B**) Representative images of pSMAD1-5-8 IHC staining of bone metastases from mice with water and BMP inhibitor treatment. Scale bars indicate 100µm for representative images. (**C**) Quantitation of pSMAD1-5-8 IHC staining of bone metastases from water (n=5) and BMP inhibitor (n=4) treated mice. Data are presented as mean ± SEM. Statistical value determined by students t-test.

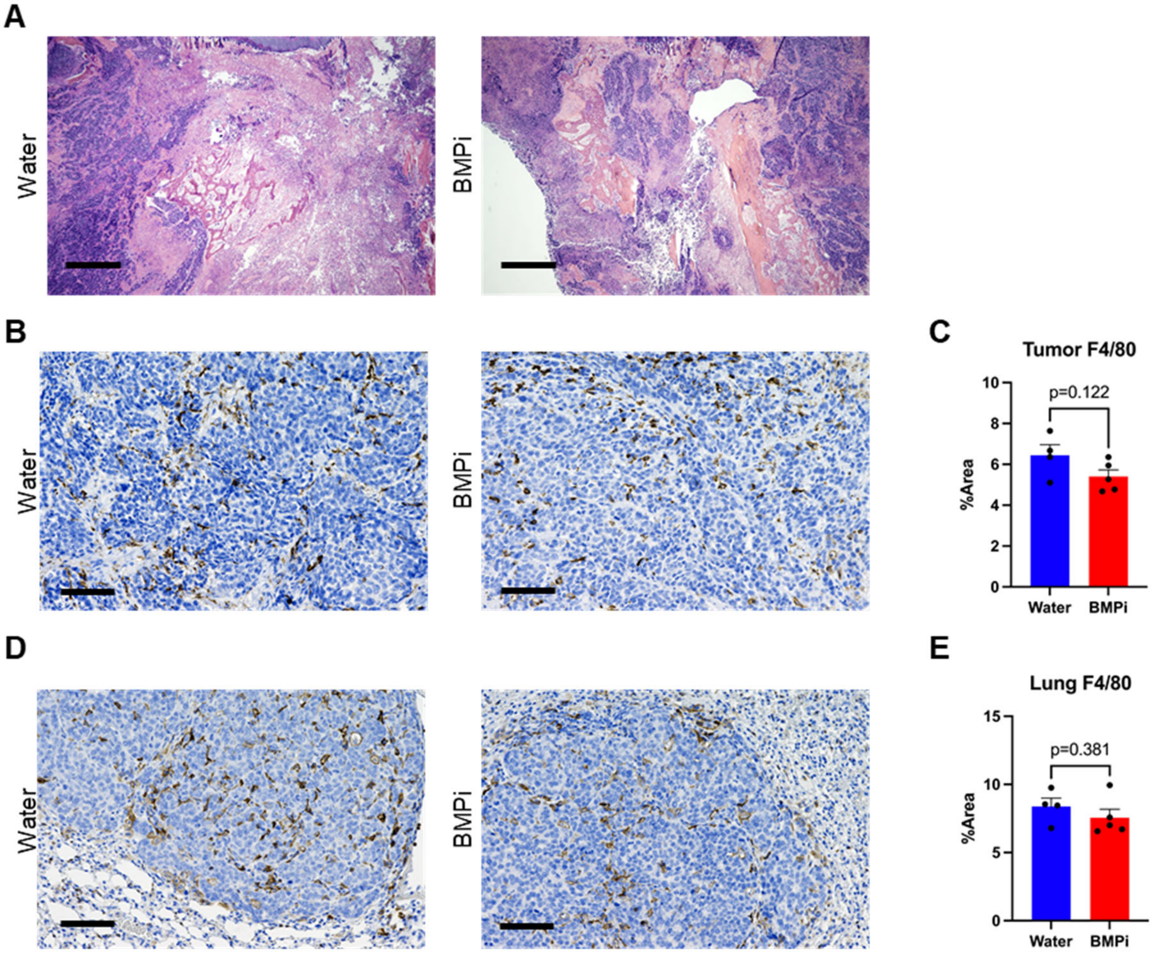

Supplemental Figure 9. Mammary Carcinoma Bone Metastases with BMP Inhibition During Trained Immunity Reprogramming. (**A**) Representative image of H&E staining of intratibial tumor from water and BMP inhibitor treated mice. (**B**) Representative images of F4/80 IHC staining of bone metastases from mice with water and BMP inhibitor treatment. (**C**) Quantitation of F4/80 IHC staining of bone metastases from water (n=4) and BMP inhibitor (n=5) treated mice. (**D**) Representative images of F4/80 IHC staining of lung metastases from mice with water and BMP inhibitor treatment. (**E**) Quantitation of F4/80 IHC staining of lung metastases from water (n=4) and BMP inhibitor (n=5) treated mice. Scale bars indicate 500µm for representative images (**A**). Scale bars indicate 100µm for representative images (**B, D**). Data are presented as mean ± SEM. Statistical value determined by students t-test (**C, E**).

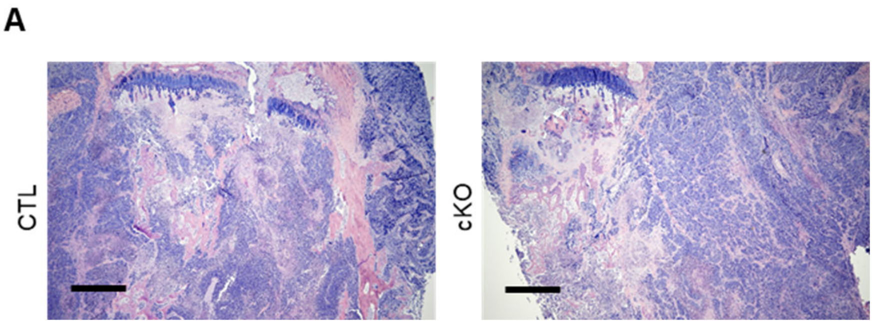

Supplemental Figure 10. Representative Images of a Transgenic Mouse Model of Mammary Carcinoma Bone Metastases. (**A**) Representative image of H& E staining of intratibial tumor from LysMCre BMPR1a CTL and cKO transgenic mice treated with β-glucan. Scale bars indicate 500μm for representative images.

Supplemental Tables:

Supplemental Table 1. Archival Metastatic Breast Cancer Bone Patient Cohort Clinical Information.

Supplemental Table 2. Differentially Expressed Genes of Metastatic Breast Cancer Bone Patient Cohort.

Supplemental Table 3. Digital Spatial Profiling PanCK ROI Counts of Metastatic Breast Cancer Bone Patient Cohort.

Supplemental Table 4. Digital Spatial Profiling CD68 ROI Counts of Metastatic Breast Cancer Bone Patient Cohort.

Supplemental Table 5. Digital Spatial Profiling CD3 ROI Counts of Metastatic Breast Cancer Bone Patient Cohort.

Supplemental Table 6. Multiplexed IHC Analysis of Myeloid Phenotypes in Metastatic Breast Cancer Bone Patient Cohort.

Supplemental Table 7. Differentially Expressed Genes of Matched Breast Cancer Tumor and Metastatic Bone Patient Cohort.

Supplemental Table 8. Multiplexed IHC Analysis of Immune Cell Phenotypes in Matched Breast Cancer Tumor and Metastatic Bone Patient Cohort.

Supplemental Table 9. Multiplexed IHC Analysis of Macrophage Activation in Matched Breast Cancer Tumor and Metastatic Bone Patient Cohort.
